# Human Corneal Stromal Stem Cell Treatment Reduces Established Opacities in Chronic Corneal Scarring

**DOI:** 10.3390/cells15070615

**Published:** 2026-03-30

**Authors:** Kira L. Lathrop, Julia T. Coelho, Christine Chandran, Syeda R. Ali, Moira L. Geary, Deepinder K. Dhaliwal, Vishal Jhanji, Mithun Santra, Gary H. F. Yam

**Affiliations:** 1 Department of Ophthalmology, University of Pittsburgh, Pittsburgh, PA 15219, USA; kira.lathrop@pitt.edu (K.L.L.); sra160@pitt.edu (S.R.A.); mithun.santra@pitt.edu (M.S.); 2 Swanson School of Engineering, University of Pittsburgh, Pittsburgh, PA 15261, USA; 3 McGowan Institute for Regenerative Medicine, University of Pittsburgh, Pittsburgh, PA 15219, USA; 4 Singapore Eye Research Institute, Singapore 169856, Singapore

**Keywords:** corneal stromal stem cells, chronic scarring, opacity volume, scar reduction, matrix metalloproteinase

## Abstract

**Highlights:**

**What are the main findings?**

- hCSSC treatment actively downsized pre-existing corneal opacities.
- Increased total MMP activity and a higher MMP2/TIMP2 expression ratio by hCSSCs under pro-inflammatory priming facilitate scar matrix degradation.

**What are the implications of the main findings?**

- Chronic corneal fibrosis/opacity can be biologically modulated by cell-based therapy.
- Treatment with hCSSCs reprograms the proteolytic activity and disrupts pathological ECM to actively reduce pre-existing fibrosis.

**Abstract:**

Corneal fibrosis, clinically referred to as corneal scarring, disrupts the normal architecture and transparency of the cornea and remains a major cause of visual impairment worldwide. Although corneal transplantation can restore vision, its effectiveness is constrained by limited accessibility, donor tissue shortages, and the risk of allograft rejection. Treatments with human corneal stromal stem cells (hCSSCs) have demonstrated scarless healing in preclinical models of acute corneal injury. Here, we report that hCSSCs also modulated pre-existing corneal opacities. We established a reproducible in vivo model of chronic corneal opacity. Given that scar severity varies among corneas even after identical injuries, we developed a non-invasive, image-based method to quantify opacity volume longitudinally in individual corneas. Using this approach, we evaluated the scar-reducing potential of three hCSSC batches previously shown to inhibit acute scarring. Following cell treatment, the pre-existing opacity volumes gradually decreased. In vitro, hCSSCs exposed to pro-inflammatory stimulus exhibited increased metalloproteinase (MMP) activity relative to tissue inhibitor of metalloproteinase (TIMP), as indicated by an elevated MMP2/TIMP2 ratio. This shift may promote matrix remodeling and scar resolution. Overall, our findings provide proof-of-concept for hCSSC-based therapy as a strategy to reduce established corneal scarring and restore corneal transparency.

## 1. Introduction

A transparent cornea is essential for normal vision; however, corneal clarity and function can be compromised by haze, clouding, and opacities resulting from trauma, chemical injury, infection, or diseases [1]. The wound-healing response and tissue remodeling processes trigger inflammation and fibrosis, leading to the deposition of abnormal, excessive, and disorganized extracellular matrix (ECM). This structural disruption produces opacities and scarring that block or distort light transmission [2]. Corneal scarring can be permanent, causing significant visual impairment or blindness. Globally, it is the fourth leading cause of blindness, affecting more than 12 million individuals (Eye Bank Association of America; https://restoresight.org/cornea-donation/; accessed on 25 February 2026). Currently, corneal transplantation remains the standard treatment to restore vision; however, its clinical utility is limited by restricted accessibility, donor tissue shortages, risk of graft rejection or failure, and post-surgical complications [3]. These limitations underscore the need for alternative therapeutic strategies that directly target fibrotic remodeling and restore corneal transparency.

Regenerative cell therapy has emerged as a promising strategy for treating corneal opacities and may offer an alternative to corneal transplantation [4,5]. Human corneal stromal stem cells (hCSSCs), the progenitors of corneal stromal keratocytes (CSKs), have demonstrated robust anti-fibrotic and pro-regenerative effects in multiple preclinical models of acute corneal stromal injury, including mechanical ablation, laser in situ keratomileusis (LASIK), alkali burns, and cryo-injury. In these settings, administration of hCSSCs soon after injury reduces fibrotic matrix deposition, suppresses myofibroblast (myoF) differentiation, and preserves stromal ultrastructure [6,7,8,9,10,11,12,13,14,15,16]. Specifically, hCSSC treatment downregulates expression of fibrosis-associated genes such as collagen type III (Col3), fibronectin (Fn), and α-smooth muscle actin (αSMA), while promoting regeneration of a native-like, lamellar collagen architecture. Functionally, CSSC-treated corneas exhibit reduced light scattering and improved transparency compared with injured controls.

Mechanistically, hCSSCs modulate both inflammatory and fibrotic signaling pathways. They attenuate early innate immune activation through tumor necrosis factor-inducible gene 6 (TSG-6)-mediated signaling, limiting neutrophil infiltration, and dampening pro-fibrotic cytokine cascades [9]. In parallel, hCSSCs secrete transforming growth factor β3 (TGFβ3), a regenerative isoform that counterbalances TGFβ1/β2-driven myoF differentiation and promotes scarless matrix assembly [17]. Beyond direct cellular activity, hCSSCs exert potent paracrine effects through exosomes and extracellular vesicles that transfer anti-fibrotic microRNAs, such as miR-29a [18], thereby repressing collagen overproduction and pathological ECM deposition in recipient cells, and attenuating fibrosis. Most recently, we demonstrated that transient pro-inflammatory priming enhanced the regenerative potency of hCSSC-derived exosomes by enriching them with full-length TGFβ3 mRNA (Santra et al., manuscript under review). Following exosome uptake, recipient stromal cells produced higher levels of functional TGFβ3 protein, reinforcing anti-fibrotic signaling and promoting tissue repair. Collectively, hCSSC-based therapy can be a promising approach to inhibit corneal fibrosis and restore corneal transparency.

Despite compelling evidence in acute injury models, most prior studies have focused on preventing fibrosis rather than reversing established scar tissue [7,11,12,19,20]. Acute models typically involve treatment shortly after injury, during a window in which myoF differentiation and ECM disorganization remain plastic and potentially repressible. While such models are valuable for studying early wound-healing responses, they do not fully reflect the clinical scenario in which patients typically present with established scarring and long-standing vision impairment. Therefore, a significant gap remains in therapies capable of resolving pre-existing opacities and addressing it is crucial for advancing clinical translation of CSSC-based therapy. In contrast to acute injury settings, chronic scarring represents a stabilized pathological state characterized by persistent inflammatory signaling, sustained myoF activity, cross-linked and disorganized ECM accumulation, and altered stromal phenotypes. These features create a self-reinforcing fibrotic niche that may resist regenerative interventions. Whether hCSSCs can reprogram this chronically fibrotic microenvironment and shift it to a regenerative state remains unknown. A central mechanistic question is whether hCSSCs actively modulate fibrosis through matrix degradation and remodeling, which is governed by the balance between matrix metalloproteinases (MMPs) and their tissue inhibitors (TIMPs). Demonstrating that hCSSC treatment can rebalance the proteolytic activity, disrupt pathological ECM structure, and restore stromal organization would represent a significant advance beyond an early fibrosis prevention.

To address this translational and mechanistic gap and better align with clinical needs, we evaluated the therapeutic potential of hCSSCs in a model of chronic corneal scarring. First, we developed a reproducible mouse model of persistent corneal opacity that recapitulates stabilized stromal fibrosis/scarring. Second, we implemented a non-invasive, image-based method to quantify corneal opacity longitudinally in individual eyes using optical coherence tomography (OCT) combined with computational morphometry. As scar severity varies among corneas even after identical injury due to inherent difference in wound-healing responses, longitudinal assessment of the same cornea at multiple time points provides greater sensitivity and interpretative rigors than end-point comparisons between grouped samples. Third, we examined whether hCSSC treatment could modulate established scar tissue by monitoring opacity volume changes over time and assessing fibrosis-associated marker expression at the final harvest. This experimental framework models a clinically relevant therapeutic window in which treatment is initiated after persistent opacity has formed. Collectively, these findings extend the paradigm of hCSSC therapy from prophylactic scar prevention to active intervention of pre-existing fibrosis, thereby strengthening its translational potential for treating patients with chronic corneal scarring.

## 2. Materials and Methods

### 2.1. A Mouse Model of Chronic Corneal Scarring

All animal procedures strictly adhered to the guidelines of The Association for Research in Vision and Ophthalmology Statement for the Use of Animals in Ophthalmic and Vision Research and the NIH regulations on the Care and Use of Laboratory Animals. The Institutional Animal Care and Use Committee of the University of Pittsburgh approved the protocol (24115664; approved 21 November 2024). Ninety-seven BALB/c mice (breeder pairs from Charles River Lab, Wayne, PA, USA), aged 10 to 12 weeks and with an approximately 1:1 male-to-female ratio, were used. Mice were housed in an AAALAC-accredited holding facility (Division of Laboratory Animal Resources, University of Pittsburgh) under a 12 h light/dark cycle with food and water available *ad libitum*. Anesthesia for surgery and ocular examination was achieved by intraperitoneal injection of ketamine (50 mg/kg; Dechra, Fort Worth, TX, USA) and xylazine (5 mg/kg; Rompun, Dechra). Local analgesia was achieved by topical proparacaine hydrochloride (0.5%, Alcon, Fort Worth, TX, USA) to the right eyes, which received procedures of corneal injury and treatment, performed by GY. Briefly, corneal epithelium (sparing the limbus) was removed using a high-speed AlgerBrush II (Accutome Inc, Malvern, PA, USA) and a #15 surgical blade. The basement membrane and anterior stroma, about 0.5 mm in diameter and 20–30 μm in depth, were ablated by a second AlgerBrush burr (Supplementary Figure S1). After rinsing with normal saline and brief drying with a sterile cotton spear, topical tobramycin ophthalmic solution (0.3%, USP, Somerset Therapeutics, Hollywood, FL, USA) was applied and continued three times daily for three days. For groups assigned two or three stromal injuries, the procedure was repeated at weekly intervals (Supplementary Figure S2). At one to four weeks after the final injury, mice were sedated with intraperitoneal ketamine and xylazine, followed by euthanasia with cervical dislocation.

Sixty corneas were randomly put into three groups assigned for single, double, and triple injuries. Group one (24 corneas) received a single injury at day 0; group two (18 corneas) received injuries at day 0 and 7; and group three (18 corneas) at day 0, 7, and 14. Corneas were harvested for scar assessment at weekly intervals after the final injury, with each time point including *n* = 6 corneas (maintaining a 1:1 male-to-female ratio) (Supplementary Figure S2). Isolated corneas were imaged against a dark smooth background. Corneal and scarred areas were measured in a blinded manner using Fiji (https://fiji.sc/, accessed on 26 February 2026), and the percentage of scarring was calculated as previously described [12,18].

### 2.2. Ophthalmic Examination and Corneal Thickness Measurement

Corneas before and at weekly intervals after injury and treatments were examined using Spectral Domain Ophthalmic Coherence Tomography (SD-OCT, Envisu R2210, Leica [Bioptigen], Morrisville, NC, USA) using linear volume scans (4 × 4 mm area, 1024 × 1000 × 100 volumes). Accurate localization of the same region on each subsequent image was achieved by locating and aligning the iris in the axial and sagittal images prior to capturing the volume image. Corneal thickness was measured by taking an average of 5 measurements obtained at the center (0 mm) and at 0.5 mm on both vertical and horizontal meridians [16].

### 2.3. OCT Image 3D Reconstruction and Opacity Volume Measurement (OVM)

**Figure 1:**
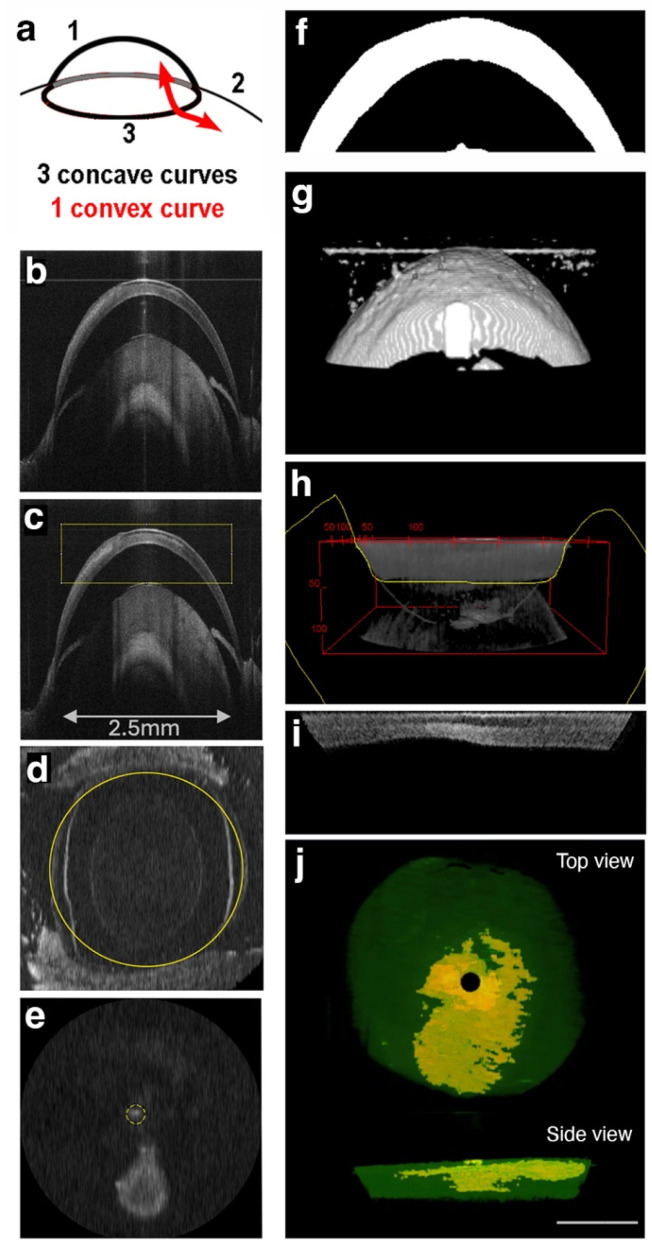
**Three-dimensional** (**3D**) **opacity volume analysis in mouse corneas.** (**a**) Isolating a cornea for 3D analysis is challenging due to its multiple curves. (**b**) OCT B-scans reveal the presence of lens and iris within the imaging field, complicating the isolation of the cornea for detailed 3D analysis. (**c**) Initial processing involved registering the B-scans to compensate for heartbeat and respiration artifacts. To begin isolating the cornea from surrounding tissues and to optimize processing time, the cornea was cropped. (**d**) Re-segmenting the volume into a coronal view enabled cropping of a standardized corneal button, ensuring consistent analysis and avoiding inclusion of the eyelids. (**e**) The characteristic specular reflection seen in OCT image was removed by defining a standard region and clearing this reflective area. (**f**) Volumes were binarized to delineate the 3D region for quantification. (**g**) The binarized volumes were examined in the 3D viewer (FIJI) and any remaining background or reflection artifacts above the corneal surface were removed. (**h**) The corneal volume was flattened, providing standard alignment of corneal layers. After flattening, tissues posterior to the cornea (iris and lens) were removed. (**i**) A dual-thresholding approach was employed: the flattened volume was firstly thresholded to define the entire corneal button, and then thresholded again to delineate scar dimensions and volume. Thresholds were determined using naïve corneas (*n* = 50) to establish normal signal intensity; values exceeding those in naïve samples were classified as hyper-reflective fibrotic regions. (**j**) Fibrotic regions were overlaid on the corresponding corneal button to illustrate the location and extent of scarring.

To quantify corneal volume with fibrosis, volumetric OCT datasets were processed and analyzed using FIJI and Python 7.3 (Python Software Foundation, 2016). The workflow is summarized in Figure 1. Isolating the cornea in OCT volumes for 3D analysis is challenging due to the multiple curves of the tissue and the presence of adjacent tissues and surface reflections (Figure 1a). To address this challenge and provide more quantitative analysis of the fibrotic region, we developed custom macros to separate the cornea from surrounding tissues and reflections, correct for heartbeat and respiration artifacts, and determine fibrotic from normal tissue. OCT volume was prepared for analysis by first standardizing the area to be analyzed. A digital corneal button was created which encompasses the anterior cornea and region of fibrosis (Figure 1c,d). The characteristic OCT specular artefact was removed by clearing a constant sized region throughout the corneal volume (Figure 1e). Any remaining background noise and reflections were manually eliminated from the corneal surface using 3D viewer in FIJI (Figure 1g), and the volume was flattened in Pyron using the corneal surface as the reference layer (Figure 1h). This provides a horizontal alignment of corneal layers within a consistent sample field across all eyes. After flattening, tissues posterior to the cornea such as those of the iris and lens were easily removed from the volume (Figure 1i).

Analysis was conducted by first applying a fixed threshold to isolate the corneal button from any background noise. The threshold level was determined by segmenting a representative sample of normal corneas (*n* > 20 samples) from the background. The average intensity of samples was used as the cutoff threshold for the segmentation macro. Overall corneal volume measurements were recorded in Excel (MS Office, version 16.106.1). Next, the normal intensity level of the signals in naïve clear corneas (*n* > 50 samples) was determined and this measurement level was used to establish a fixed threshold cutoff to distinguish normal from pathological opacity signals. Regions with signals higher than this naïve-defined threshold represented areas of increased optical density, which is characteristic of hyperreflective fibrotic tissues in scarred corneas (Figure 1j). The opacity volume was also recorded in Excel. Supplementary Figure S12 demonstrates that the region with increased OCT signals corresponded to the immunoreactive signals of αSMA and fibronectin expression (fibrosis markers) on the same scarred cornea.

This dual-threshold approach allows us to distinguish between total corneal volume and fibrotic regions. By analyzing volumetric data rather than single slices, this method accounts for the variability in scar tissue location and provides a detailed 3D representation of both the entire cornea and scarred regions.

### 2.4. Validation of Opacity Volume Assessment

Two masked raters independently performed identical protocols of image processing, segmentation, and dual-threshold analysis to measure corneal and scar volumes on 12 sets of OCT-captured corneal images, including six with indistinct opacities and six with visible scarring. This sampling approach encompassed a range of scar scenarios. To account for variation in the depth of corneal buttons, the percentage of scar volume relative to corneal volume was calculated. The process was repeated after 48 h. Inter- and intra-rater reliability and agreement were assessed using the intraclass correlation coefficient (ICC, SPSS IBM version 28.0.1.1) with a 95% confidence interval, by applying a two-way random-effects absolute agreement model. Values < 0.5 indicate poor agreement, 0.5 to 0.75 fair, 0.75 to 0.9 good, and >0.9 excellent.

### 2.5. Donor Corneas, Human CSSC Isolation, and Culture

This study received approval from Human Stem Cell Research Oversight (hSCRO Protocol# 202300049; approved 17 February 2025) at the University of Pittsburgh. All procedures complied with the Declaration of Helsinki, and the research protocol was approved by the Committee for Oversight of Research and Clinical Training Involving Decedents (CORID) (Protocol #161; approved 12 September 2024), University of Pittsburgh. Human corneas approved for research purposes were obtained from de-identified donors younger than 60 years old through the Center for Organ Recovery and Education (Pittsburgh, PA, USA) and Eversight Eye Bank (Cleveland, MI, USA) with informed consents obtained from next of kin (see Supplementary Table S1 for donor information). Donors had no history of ocular diseases or cancer and tested negative for transmissible diseases, including HIV, hepatitis B and C, and syphilis. Corneas were free from injuries or inflammation and had corneal endothelial cell counts greater than 2500 cells/mm^2^. All tissues were harvested by trained eye bank technicians, preserved in Optisol GS (Bausch & Lomb Inc., Rochester, NY, USA), and used within 10 days.

After removing corneal epithelium, iris, and endothelium, the anterior limbal stroma (about 0.5 mm wide and 100 μm deep) was isolated and digested with collagenase A (1.2 mg/mL; Roche, Indianapolis, IN, USA) for 6 to 8 h at 37 °C, according to previously published protocols [12,18]. The resulting single cells were cultured in stem cell growth medium (JM-H) supplemented with 2% (vol/vol) pooled human serum (Innovative Res, Novi, MI), on FNC-coated tissue culture polystyrene (FNC mix from Athena Enzyme Systems, Baltimore, MD, USA) [7,21]. At passage 3, the cells were characterized for clonal formation efficiency, cell growth by xCelligence, and stem cell homogeneity (CD73^high^ ALDH3A1^+^ CD31^neg^ CD45^neg^) by flow cytometry (Supplementary Information) [15].

### 2.6. hCSSC Treatment to Mouse Corneas with Chronic Scarring

Thirty-seven Balb/c mice with chronic corneal scarring created one-week after double injuries were anesthetized. Prior to treatment, OCT imaging was performed to record baseline scar levels. The eyes were randomly assigned to receive one of three CSSC treatments (HC436, HC439, and HC728; each with previously reported scar-inhibitory effects) [12,13]. Corneal epithelium was removed using an Algerbrush, after which the corneal surface was rinsed with normal saline and briefly dried. The wound surface was treated with 0.5 μL fibrinogen (37 mg/mL, Sigma-Aldrich, St Louis, MO, USA) containing 1 × 10^5^ CSSCs (a cell dosage double that is used in acute injury models), followed by 0.3 μL thrombin (100 U/mL, Sigma-Aldrich) (Supplementary Figure S1). Control groups included fibrin only and untreated scarred corneas. Each group consisted of at least six corneas. TobraDex ophthalmic eye drops (Alcon) were administered three times daily during the first week, twice daily in the second week, and once daily in the third week. Corneas were examined and imaged weekly using SD-OCT.

### 2.7. Gene Expression

Isolated corneas were lysed in RLT buffer (Qiagen, Germantown, MD, USA) freshly added with 1% β-mecaptoethanol (β-ME) and disrupted with MagNA Lyser beads in a MagNA Lyser instrument (Roche). Alternatively, cell samples were collected in RLT buffer with β-ME. Total RNA was extracted using RNeasy Miniprep kit (Qiagen) with on-column RNase-free DNase kit (Qiagen), following manufacturers’ instructions. RNA was quantified using Nanodrop One (Thermo Fisher Sci, Waltham, MA, USA), and 500 ng RNA was reverse transcribed to cDNA using SuperScript III Reverse Transcriptase kit and random primer hexanucleotides (10 ng/mL, Thermo Fisher Sci). Target gene expression was assessed using specific primers (Table S2) and SYBR Green Real-Time Master Mix (Applied Biosystems, Thermo Fisher Sci) in a QuantStudio3 Real-Time PCR System (Applied Biosystems). Experiments were done in triplicate, the relative RNA abundance was determined by 2^−ΔΔCt^ method after normalization with housekeeping 18S, and fold changes were presented as mean ± SD. Significance was determined by the non-parametric Mann–Whitney U test (Prism 10).

### 2.8. Whole Mount Immunofluorescence

Corneas were fixed in freshly prepared neutral-buffered 4% paraformaldehyde overnight at 4 °C and washed with PBS 3 to 4 times. They were treated with ice-cold 50 mM NH_4_Cl/PBS to quench aldehyde residues. After PBS washes, samples were permeabilized and non-specificities were blocked with PBS containing 0.15% saponin (Sigma), 1% bovine serum albumin (BSA), and 1.5% normal goat serum (Gibco) for two hours at room temperature. Without washes, they were incubated with AlexaFluor488-conjugated anti-αSMA (Invitrogen, Carlsbad, CA, USA), rabbit anti-mouse fibronectin antibodies (Abcam, Cambridge, UK), and DAPI (4′,6-diamidino-2-phenylindole) (Supplementary Table S3). After PBS washes, samples were mounted with Immu-mount (Thermo Fisher) and examined under confocal microscopy (Fluoview 1200, Olympus, Center Valley, PA, USA).

### 2.9. Mouse Macrophage Culture, M1 and M2 Polarization, and Medium Conditioning

Mouse RAW264.7 cells (American Type Cell Collection ATCC, Manassas, VA, USA) were treated with lipopolysaccharide (LPS, 5 ng/mL, Sigma-Aldrich) for 48 h to induce pro-inflammatory M1 polarization [18]. Alternatively, cells were treated with interleukin-4 (IL-4, 10 ng/mL, Sigma-Aldrich) for 48 h to induce anti-inflammatory M2 phenotype. After washing, M1, M2, and control RAW (M0) cultures were replenished with plain culture medium (DMEM/F12) supplemented with 1% insulin–transferrin–selenium and antibiotics (DM+) and incubated for 48 h. Conditioned media (CM) were collected and concentrated to 1/20th of the original volume using a MicroCon centrifugal filter (YM-10, MilliporeSigma, Burlington, MA, USA). Total protein content was determined using the Pierce BCA Protein assay (Thermo Fisher).

### 2.10. MMP Activity Assay

hCSSCs were cultured to 70% confluence in a 6-well plate and treated with RAW CM concentrates (500 μg protein per well). After 48 h, the cultures were washed, replenished with DM+, and incubated for an additional 48 h. Culture supernatants, representing the extracellular (EC) fractions, were collected and centrifuged at 400 g for 5 min to remove debris. Samples were concentrated using a YM-10 filter to about 1/20th of their original volume. Simultaneously, the cells were washed and lysed in RIPA buffer (Radioimmunoprecipitation assay; Thermo Fisher Sci) added with protease inhibitor cocktail (Complete^TM^, Roche) and phenylmethylsulfonyl fluoride (PMSF). After sonication, the lysates were centrifuged at 32,000× *g* for 15 min at 4 °C. The resulting clear supernatant was collected as the intracellular (IC) soluble fractions. Total protein content was determined using Pierce BCA Protein assay. Total MMP activity in both EC and IC fractions (5 μg protein) was measured using a fluorescence-based EnzChek gelatinase/collagenase assay (Molecular Probe, Thermo Fisher Sci), according to manufacturer’s instructions. Assay specificity was confirmed by the addition of an MMP inhibitor, 1,10-phenanthroline monohydrate. MMP activity was quantified by the enzymatic digestion of fluorescein-conjugated DQ^TM^ gelatin to yield high fluorescence peptides, which were measured using a SpectraMax M3 multiplate reader (Molecular Devices; San Jose, CA, USA) at wavelengths of excitation at 495 nm and emission at ~515 nm.

### 2.11. MMP and Tissue Inhibitors of Metalloproteinase (TIMP) Gene Expression

CSSC lysates after treatments with M0, M1, and M2 RAW CM, respectively, were evaluated for expression changes of human MMP2, MMP9, TIMP1, and TIMP2 (specific primers in Supplementary Table S2). The 18S-normalized values were compared, and fold changes were calculated. Expression ratios of MMP2/TIMP2 and MMP9/TIMP1 were obtained to compare M0, M1, and M2 RAW CM effects on CSSCs.

### 2.12. Statistics and Sample Size Calculation

Mouse experiments were performed using a minimum of 6 corneas per group. The sample size was calculated using G*Power 3.1, *n* = 1 + 2 C (s/d)^2^, where α is significance level and power (1-β) [22]. The constant C is 10.51 with *p* at 0.05 (α) and power (1-β) at 0.8. The effect size and SD are estimated to be 30% and 20%, respectively, giving *n* = 6 per group. All experiments were done in triplicate, and data presented as mean ± SD. Mean value was compared using unpaired Student’s *t*-test or ANOVA with a post hoc Bonferroni test using GraphPad Prism 10. Percentage changes in scar volume were compared across groups using the Kruskal–Wallis test, followed by Dunn’s post-hoc test for multiple comparisons. Non-parametric comparison was conducted with Mann–Whitney U test. *p* < 0.05 was considered statistically significant.

## 3. Results

### 3.1. Repeated Mechanical Injury Generated Chronic Corneal Scarring in Mice

**Figure 2:**
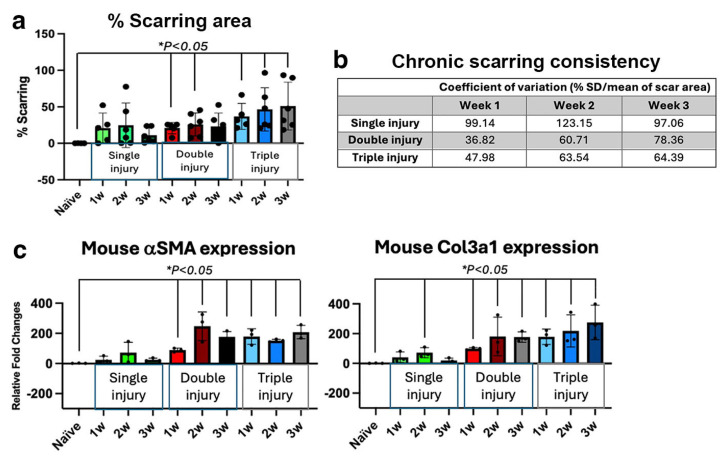
**Development of a chronic corneal scarring model**. Mouse corneas received anterior stromal injury by a single mechanical ablation at time 0 or multiple ablations at consecutive weeks. The corneas were collected at different weeks post final injury to examine scar formation (percentage of scarring area and expression of fibrosis-related genes). (**a**) Double and triple injuries consistently induced scarring with significant difference from naïve corneas, whereas a single injury showed variable scarring outcomes. (**b**) Consistency of scarring was indicated by the coefficient of variation (CoV, percentage of SD/mean) of scar area. Corneas at 1-week post-double injury exhibited the smallest CoV (36.8%) when compared with >50% in corneas with other injury frequency and collection time points. (**c**) Significant upregulation of mouse fibrosis-related genes (αSMA and Col3A1) in corneas after multiple injuries. * *p* < 0.05, comparing different injury groups (*n* = 6) with naïve control (*n* > 8); non-parametric one-way ANOVA.

Mouse corneas subjected to mechanical stromal ablation twice in consecutive weeks (*n* = 18 corneas) and three times within three weeks (*n* = 18 corneas) developed visible scarring in all corneas harvested at 1 to 3 weeks following the final ablation (Supplementary Figure S3). In contrast, among 24 corneas that received a single injury, scarring was variable over four weeks post-surgery. In randomly selected groups of six corneas per harvest time point, scarring was observed in four corneas at week 1, five at week 2, four at week 3, and three at week 4. These results indicate that a single anterior stromal injury did not consistently induce persistent scarring, whereas repeated injuries reproducibly generated stromal scarring lasting at least three weeks post-injury. Quantitative scar area analysis showed that the percentage of scarring increased progressively following double and triple injuries and was significantly greater than in naïve corneas (Figure 2a). Corneas harvested one week after double injury exhibited the lowest coefficient of variation (CoV, percentage of SD/mean) in scar area (36.8%) compared with other time points (>50%) (Figure 2b). In contrast, single injury produced highly variable scarring, with CoV values three to four folds higher than those observed one week after double injury. Tissue RNA analysis further demonstrated progressive upregulation of fibrosis-associated genes (αSMA and Col3a1) at all time points after double and triple injuries (*p* < 0.05, Mann–Whitney U test) while the gene expressions varied in samples with single injury (Figure 2c). Collectively, these findings demonstrate that mechanical ablation injury for two times or more at consecutive weeks reliably induced chronic stromal scarring in mouse corneas, whereas a single injury yields transient opacities that fluctuated within 3 to 4 weeks. Based on these results, we adopted a double mechanical ablation injury model to consistently generate chronic stromal scarring prior to cell treatment in this study.

### 3.2. Validation of Opacity Volume Measurement (OVM) Assay

Corneas before and at weekly intervals after double injury were examined using SD-OCT. To quantify opacity changes, volumetric OCT datasets from a total of twelve mouse corneas (six corneas with indistinct opacity and six corneas with visible scarring) were processed for corneal and scar volume measurements. Two masked raters independently performed image processing and measurements with identical protocols. The data were calculated for the percentages of scar volume within the respective cornea.

Using intraclass correlation coefficient (ICC) assay, the inter-rater reliability was 0.999 with a confidence interval of 0.997–1.0 (Table 1a). These results are considered excellent. Intra-rater reliability was also excellent, with ICC values for the first rater of 0.997 and a confidence interval of 0.989–0.999 (Table 1b). The consistency of the second rater was also excellent, with an ICC of 1.00 (Table 1c).

### 3.3. Donor CSSCs with Scar Inhibitory Effect on Acute Stromal Wounds in Mouse Corneas

We tested three donor CSSC batches (HC436, HC439, and HC728), which inhibited corneal scarring in a mouse model of acute stromal ablation wounds, as reported in our studies [13,15]. In culture, the stellate-shaped cells exhibited proliferative capability with cell doubling time of 28.1 ± 1.2 h measured by xCelligence (Supplementary Figure S4). By flow cytometry, they showed a characteristic phenotype of CD73^high^ ALDH3A1^+^ CD31^neg^ CD45^neg^. In vivo, topical hCSSC treatment to acute stromal wounds in mice inhibited more than 50% scarring compared with untreated injured controls (data from [15]) (Supplementary Figure S5). The percentages of scar inhibition were 80.1 ± 16.6% after HC436 treatment, 95.8 ± 1.3% after HC439 treatment, and 73.5 ± 6.3% after HC728 treatment.

### 3.4. hCSSC Treatment Reduced Chronic Opacities in Mouse Corneas

**Figure 3:**
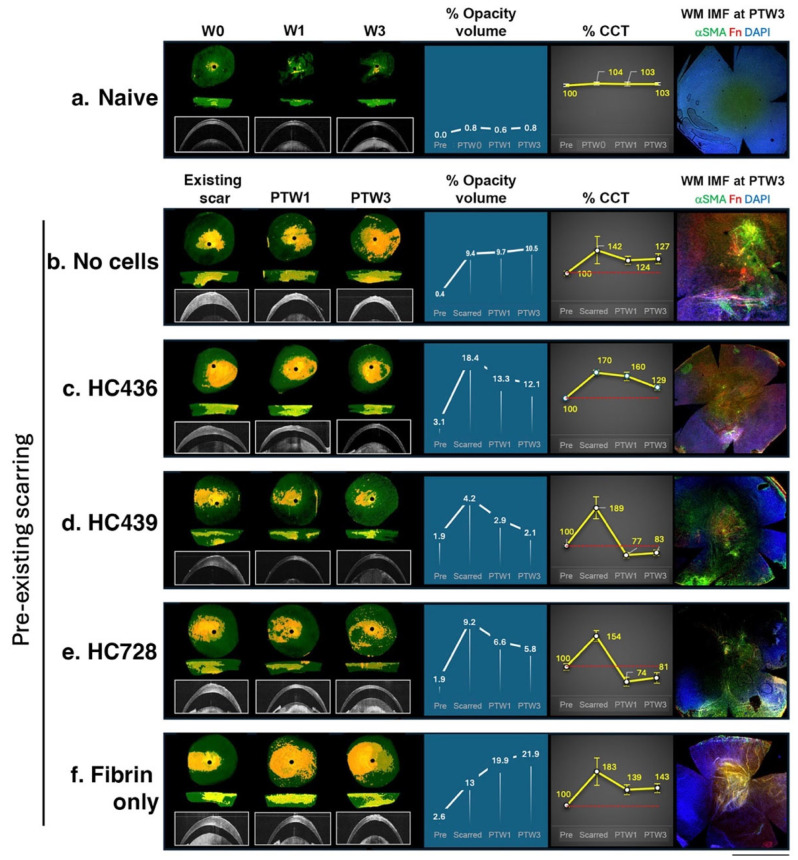
Representative corneal images illustrating scar modulation after CSSC treatments (HC436, HC439, and HC728) (**c**–**e**), compared with controls (naïve, untreated scarred, and fibrin-only treatment) (**a**,**b**,**f**) (*n* > 6 each group). Opacity volume analysis showed gradually reducing % opacity volume in scarred corneas after CSSC treatment up to post-treatment week (PTW) 3, whereas increased opacity was detected (**c**–**e**) when corneas were treated with fibrin-only (**f**) or without treatment (**b**). Minimal opacity was detected in naïve corneas from week 0 to 3 (W0 to W3) (**a**). Cell treatments also reduced central corneal thickness (% CCT). End-point analysis with whole-mount immunofluorescence (WM IMF) revealed a downregulated expression of fibrosis markers (a-smooth muscle actin αSMA and fibronectin Fn) after cell treatments but not in scarred controls. Scale bar: 1 mm.

To determine whether hCSSCs previously shown to possess scar-inhibitory properties could modulate pre-existing corneal scarring, we utilized the double ablation injury model that induced chronic stromal scarring (established in Section 3.1). One week after the second injury, SD-OCT imaging confirmed the presence of established opacity. Scar volume was quantified using the OVM assay prior to treatments (Figure 3). Corneas having greater than 5% opacity (compared with the pre-injured state) were selected for treatment evaluation. Thirty-seven scarred corneas were randomly assigned to receive treatment with three different hCSSC batches (HC436, HC439, and HC728), sham treatment, or remain as untreated scarred controls, with a minimum of six corneas per group.

hCSSCs (1 × 10^5^ cells) suspended in fibrin gel were applied directly onto the stromal scar surface following epithelial debridement, ensuring full coverage of the scarred region. Weekly SD-OCT imaging and OVM analysis showed that hCSSC treatments progressively reduced corneal hyperreflectivity over a three-week period (Figure 3). Treatment with HC436 reduced opacity in five of seven treated corneas at post-treatment week 3 (PTW3), with an average reduction of 34.1 ± 8.9% relative to the pre-treatment levels; the remaining two showed minimal differences (Figure 3c, Supplementary Figure S6). Among HC439-treated corneas, five of six exhibited a mean opacity reduction of 43.8 ± 13% (Figure 3d, Supplementary Figure S7). Mean CCT in these corneas decreased to levels comparable to those measured prior to injury. In the HC728-treated group (*n* = 7), four corneas showed an average opacity reduction of 17.9% at PTW3 (Figure 3e; Supplementary Figure S8). The remaining three corneas displayed a modest decrease in opacity at PTW1 but returned to near pre-treatment levels by later time points. In contrast, all control corneas, either injury-only (*n* = 7) (Figure 3b) or fibrin-treated (*n* = 6) (Figure 3f), maintained persistently elevated opacity volume and CCT compared with corneas at the naïve state (Figure 3a, Supplementary Figures S9 and S10). The degree of scarring in both control groups was significantly greater than that observed in hCSSC-treated corneas (*p* = 0.001, Chi-square test).

**Figure 4:**
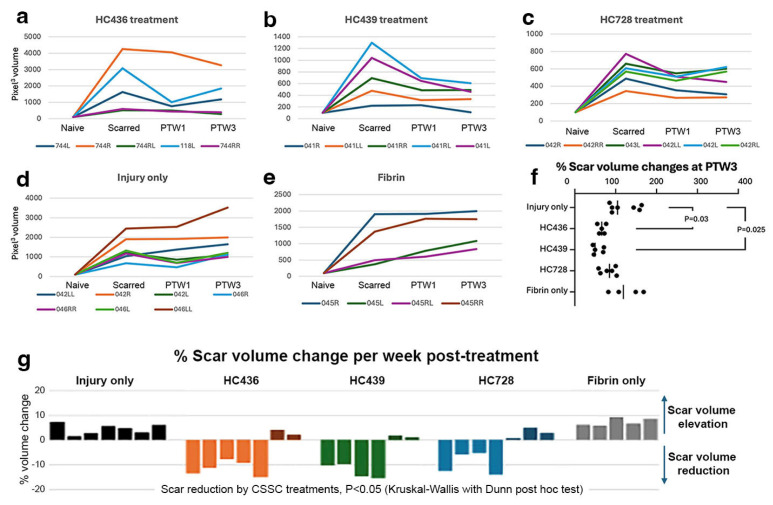
**Corneal opacity volume changes after CSSC treatments.** Mouse scarred corneas treated with hCSSCs (HC436, HC439, and HC728) exhibited gradual opacity reduction (**a**–**c**) while corneas with fibrin only (**e**) and which were untreated (**d**) displayed increased opacities. (**f**) At post-treatment week 3 (PTW3), the mean opacity volume was significantly reduced in the scarred corneas treated with HC436 and HC439, when compared with the untreated controls (*p* < 0.05, one-way ANOVA). (**g**) The weekly percentage changes of scar volume showed that CSSC-treated corneas exhibited a preferential scar reduction, which was not detected in controls (both untreated injured and fibrin-only groups).

The changes of opacity volume between hCSSC-treated and control groups is summarized in Figure 4. Both injured and fibrin-only groups showed persistently higher opacity levels, with all corneas displaying progressively intensified opacification (Figure 4d,e). On the contrary, CSSC-treated corneas showed reduced opacity from the peak level before treatment to the end-point at PTW3. Compared with pre-treatment levels, 14 out of 20 hCSSC-treated corneas (70%) showed a gradual reduction of opacity volumes (Figure 4a–c).

Both HC436 and HC439 significantly reduced scar volume at PTW3 compared with untreated scarred corneas (*p* < 0.05, Mann–Whitney U test) (Figure 4f). To further evaluate scar-reducing efficacy, weekly changes in opacity volume were analyzed. Treatment with HC436 resulted in a mean weekly scar reduction of 11.4 ± 3%, HC439 had a reduction of 10 ± 8.6%, and HC728 produced a modest reduction of 3.6 ± 6.6%. In contrast, untreated scarred controls showed an average weekly progression in scar volume of 9.7 ± 8.5%, while fibrin-only treatment had a 7.6 ± 2.7% increase (Figure 4g). Multiple comparison analysis confirmed that weekly opacity volume changes in hCSSC-treated corneas were significantly different from those in control groups (Figure 4g). Although a subset of CSSC-treated corneas did not demonstrate measurable scar reduction, they exhibited only minimal increase in opacity volume, indicating that hCSSCs did not exacerbate corneal scarring. Individual corneas in response to treatments are presented in Supplementary Figures S6–S10.

Mouse corneas harvested at PTW3 were processed for wholemount immunofluorescence to evaluate the expression of fibrosis-associated markers. Confocal images revealed that scarred corneas treated with fibrin-only strongly expressed αSMA and Fn at the scarred region, in contrast to the negligible expression in naïve controls. Significant downregulation of both markers in hCSSC-treated corneas supported the scar-reducing effect of cell treatment on this chronic fibrosis model (Supplementary Figure S11).

### 3.5. Cell Application Quality Affected Treatment Outcomes

**Figure 5:**
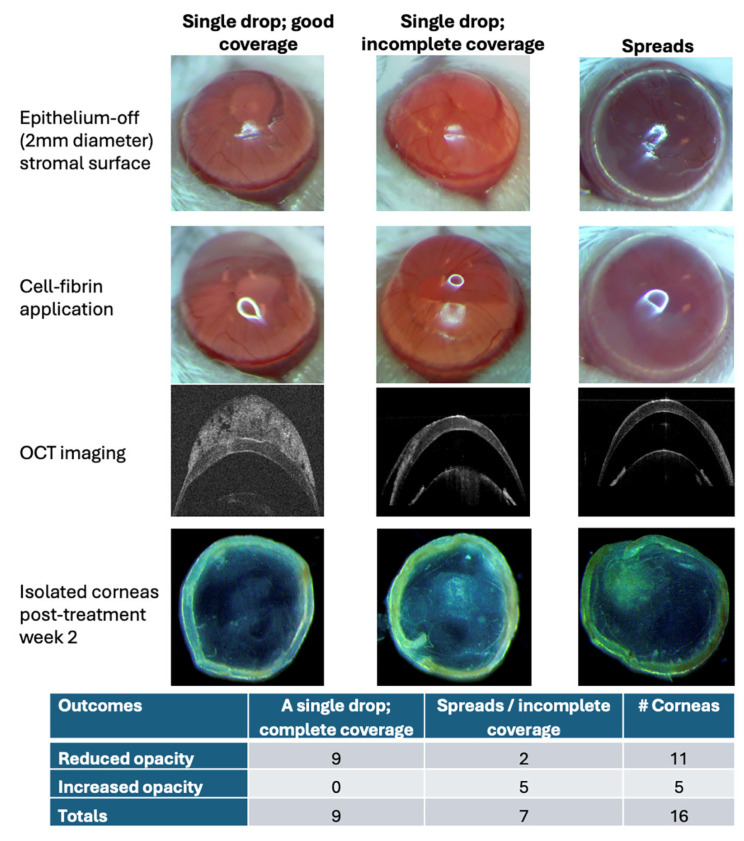
**Application quality of cells in fibrin and treatment outcomes in acute wound model.** The formation of a single drop of fibrin containing CSSCs (HC436 and HC439) covering the scarred region achieved effective scar prevention, whereas cell suspension spread over the corneal surface or partially covering the scarred region did not modulate the opacity development.

We hypothesized that the quality of hCSSC application on wound surface could affect scar modulation. In this investigation, we conducted a retrospective analysis using 16 acutely wounded corneas applied with HC436 and HC439 cells [15]. We categorized the quality of cell delivery based on whether the fibrin gel formed a discrete drop or spread as a thin layer on the corneal surface (Figure 5). At PTW2, we observed that the therapeutic outcome was associated with the mode of cell application. A single, well-formed fibrin drop containing CSSCs that adhered to and fully covered the scar region was considered a good application, and was found to consistently prevent scarring. In contrast, applications in which the cell–fibrin mix spread across the corneal surface, extended to the sclera or eyelids, or partially covering the scarred region were classified as poor and provided inconsistent cell delivery (Figure 5). Corneas receiving these CSSC–fibrin spreads or incomplete coverage showed higher levels of opacities at PTW2.

### 3.6. hCSSCs Exhibited an Upregulation and a Release of Active Matrix Metalloproteinases (MMPs) Under M1 Pro-Inflammatory Milieu

ECM degradation is closely regulated by collagenases, which are key enzymes involved in breaking down collagen during tissue remodeling [23,24]. To assess the impact of hCSSC treatment on opacity reduction, we hypothesized that therapeutically functional hCSSCs, when applied to stromal wounds in a pro-inflammatory environment, may exhibit collagenase activity, leading to alterations in collagen and ECM turnover.

**Figure 6:**
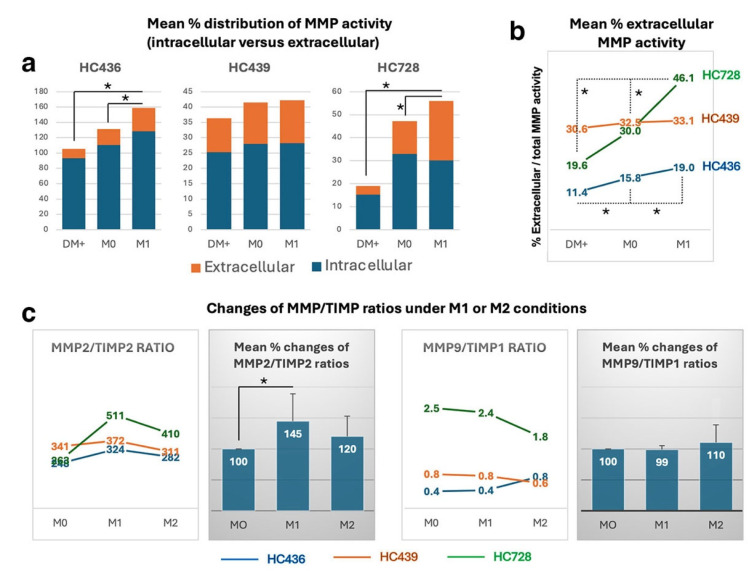
**Pro-inflammatory M1 induction of MMP expression and activity in hCSSCs.** (**a**) Increases of total MMP activity in both intracellular (cell lysate, blue) and extracellular (supernatant, orange) fractions. DM+: cells treated with plain medium only; M0: cells treated with unstimulated RAW CM; M1: cells treated with LPS-stimulated RAW CM. (**b**) Increases of secreted MMP activity by M1 treatment. (**c**) Changes of MMP/TIMP expression ratios. M1 RAW CM treatment on hCSSCs induced higher MMP2/TIMP2 ratios than samples stimulated by M2 RAW CM. The mean percentage changes of MMP2/TIMP2 ratios calculated with all hCSSC batches showed a significant increase after treatment with M1 RAW CM. In contrast, no clear differences were detected for MMP9/TIMP1 ratios. * *p* < 0.05, non-parametric one-way ANOVA.

Our previous work demonstrated that donor CSSCs exposed either to mouse RAW264.7 macrophages induced to the M1 pro-inflammatory phenotype or to a corneal stromal wound with a pro-inflammatory response, exhibited increased expression of TGFβ3 [17]. In the current study, we used a similar approach by treating hCSSCs with conditioned media (CM) from M1-stimulated RAW cell culture. Control groups included cells treated with CM from M0 RAW culture and sham medium only. After 48 h of incubation and thorough washes to remove RAW CM, CSSC cultures were replenished with basal medium for an additional 48 h. Culture supernatants and cell lysates were then collected to measure total MMP activity using the EnzChek gelatinase/collagenase assay kit. As shown in Figure 6a, all hCSSC batches exhibited increased total MMP activity, both intracellularly (cell lysates) and extracellularly (supernatants), following M1 RAW CM treatment compared with M0 and sham controls. Notably, secreted MMP activity was observed in all three CSSC batches (Figure 6b, Supplementary Table S5). Compared with M0 RAW medium (representing a naïve condition), M1 RAW CM (pro-inflammatory) increased the mean secreted MMP activity by 2.5 folds in HC436, 27.3% in HC439, and 6.9 fold in HC728 (orange bars in Figure 6a).

We next examined the expression ratios of MMP and their tissue inhibitors (TIMPs), as these ratios influence whether wound healing proceeds normally or results in excessive tissue fibrosis [25,26]. We specifically assessed the MMP2/TIMP2 and MMP9/TIMP1 ratios, which are biomarkers for various conditions such as adenomyosis, renal fibrosis, diabetic wound healing, and cancers [27,28,29,30]. Our results show that treatment with M1 RAW CM consistently elevated MMP2/TIMP2 ratios across CSSC batches, averaging 45% higher than M0 and 21% higher than RAW CM collected following M2 induction for the anti-inflammatory phenotype (Figure 6c). In contrast, the MMP9/TIMP1 ratio, recognized as a biomarker for Alzheimer’s disease, chronic obstructive pulmonary disease, and stroke [31,32,33], remained unchanged across different RAW CM treatment conditions.

## 4. Discussion

This study demonstrates that hCSSC therapy actively reduced pre-existing corneal opacities in a mouse model of chronic stromal fibrosis. These findings extend the therapeutic scope of hCSSCs beyond a prophylactic inhibition of acute haze [7,12,15] and establish proof-of-concept that pre-existing opacity—long considered largely irreversible without corneal transplantation—can be biologically modulated. This conceptual shift from scar prevention to opacity resolution represents a critical advance for translational regenerative medicine in corneal scarring disorders.

The clinical burden of chronic corneal scarring significantly outweighs that of early-stage injuries amenable to prophylactic intervention. While dense scars along the visual axis inevitably necessitate surgical removal and corneal transplantation, many patients experience mild-to-moderate opacities that impair vision but do not require immediate surgery. Even partial improvement of these opacities can allow for meaningful visual rehabilitation through non-surgical means, such as rigid gas-permeable lenses, which correct irregular astigmatism by providing a uniform refractive interface, and can postpone or alleviate the need for surgery [34]. This strategy helps conserve scarce donor corneal tissues for the most needed cases and reduces surgical wait time. This distinction is particularly important in low-resource settings, where access to donor tissue, eye banking, surgical infrastructure, and postoperative care is limited. Consequently, a scalable, cell-based approach capable of remodeling existing scar tissue could address a significant unmet global health need.

Corneal fibrosis is not a static accumulation of scar matrix but an actively maintained pathological microenvironment. Following stromal injury, quiescent keratocytes activate to become stromal fibroblasts (SFs) and myofibroblasts (myoFs) under sustained pro-inflammatory and TGFβ1/β2 signaling [35,36]. These activated cells lose keratocyte features while they proliferate and deposit excessive and disorganized ECM, including collagen types I, III, IV, and XII. Persistent basement membrane disruption and chronic cytokine exposure support myoF survival and continued matrix deposition, establishing a self-reinforcing fibrotic niche. Over time, this fibrotic matrix becomes structurally and biochemically stabilized, and it may take months to years to resolve or remain persistent [37]. Native keratocyte-derived MMPs, which normally maintain stromal homeostasis [38,39], appear insufficient to degrade the aberrantly organized ECM characteristic of chronic scars. Thus, effective treatment requires more than suppressing fibrosis; it needs to reactivate matrix turnover and destabilize pathological ECM architecture.

Various studies have established that hCSSCs suppress acute fibrosis through immunomodulatory and paracrine mechanisms, including upregulation of TSG6, secretion of TGFβ3, and delivery of anti-fibrotic microRNAs, such as miR-29a, via extracellular vesicles [9,12,13,15,17,18,40]. These pathways inhibit myoF differentiation, reduce pro-fibrotic gene expression, and promote regenerative matrix assembly. However, prevention of early fibrotic signaling does not equate to reducing mature scar tissue.

Here, we provide evidence that hCSSCs can reprogram a chronic fibrotic microenvironment. Scar reduction was found to be potentially associated with increased total MMP activity and a higher MMP2/TIMP2 expression ratio following pro-inflammatory priming. Consistently elevated MMP2/TIMP2 ratios were detected across hCSSC batches with an average of 45% increase after M1 priming over sham controls. This finding is mechanistically significant. MMP2 (gelatinase A) primarily degrades type IV and V collagens, fibronectin, laminin, and other ECM components enriched in fibrotic matrix [41]. Its activation depends on a trimeric complex involving MT1-MMP (MMP14) and TIMP2, in which TIMP2 serves as a molecular adaptor at controlled concentrations [42,43]. The MMP2/TIMP2 ratio therefore reflects the functional balance between matrix degradation (by MMP2) and inhibition (by TIMP2), whereas higher MMP2/TIMP2 ratios promote ECM degradation in tissues, like heart, and kidney [44,45]. This association indicates a reduction of fibrosis, although the specific impact of this ratio varies by disease context.

Our data suggest that the scar-reducing effect of hCSSC therapy may be associated with a shift in stromal proteolytic balance toward active matrix remodeling. Notably, this response appears to be enhanced under pro-inflammatory stimulation, indicating that hCSSCs adapt to, rather than are impaired by, the inflammatory niche. We propose that hCSSCs may function as context-responsive modulators that sense inflammatory cues and initiate coordinated resolution programs, including protease activation for stromal matrix remodeling (as proposed in this study). These activities complement previously reported mechanisms, including the production of regenerative cytokines such as TGFβ3 [17,46] and paracrine delivery of anti-fibrotic microRNAs [18]. Such an integrated remodeling response may destabilize accumulated ECM, facilitate myoF regression, and permit partial re-establishment of stromal organization compatible with reduced opacity. Although complete architectural restoration was not achieved, the observed partial reduction of opacity suggests that chronic scar tissue retains a degree of biological plasticity that can be therapeutically exploited. At the same time, the persistence of residual scarring indicates that some matrix alterations have non-repairable structures. Future studies investigating MMP inhibition, e.g., using pharmacological inhibitors such as 1,10-phenanthroline monohydrate (as applied in our in vitro assay) or conditional genetic knockdown, will be important to further delineate the mechanistic contribution of MMP-mediated remodeling to corneal scar reduction. Additionally, further characterization of the immune microenvironment in scarred corneas following hCSSC treatment will provide more mechanistic insight into the therapeutic effect of these cells.

Our results show that therapeutic scar reduction by hCSSCs was observed in the majority, though not all, of treated corneas. The opacity volume was markedly decreased in 63% of HC436-, 71% of HC439-, and 67% of HC728-treated corneas. These changes observed in a majority of examined corneas indicate the predominant therapeutic effect of treatment with a particular donor cell batch rather than reflecting technical variations. In addition, these findings underscore the potential influence of local microenvironmental factors. Variability in oxygenation, inflammatory milieu, ECM density, and cross-linking may modulate cellular viability and remodeling efficiency [47,48]. Although detailed analysis of wound physiology is beyond the scope of this study, we acknowledge that further investigation into these areas will be important for developing more comprehensive therapeutic strategies. Our pilot data further showed that fibrin gel encapsulation did not impair CSSC viability, with over 85% of cells remaining viable after 48 h in fibrin gel (Supplementary Table S4). When applied to mouse corneas, the fibrin gel is designed to dissolve within 1 to 2 days and have complete fibrinolysis in a week [49]. This time frame enables cell delivery to wound ECM for the corrective activities. The approach also provides an effective window for cell–matrix interaction while avoiding prolonged scaffold persistence. This delivery strategy is clinically translatable and adaptable to outpatient settings. Notably, spatial delivery proved critical. A focal application of a cell–fibrin drop directly over de-epithelialized scar tissue yielded superior outcomes compared with broader surface spreading. These findings highlight the importance of targeted stromal engagement and suggest that optimizing delivery geometry may significantly enhance therapeutic consistency.

A key consideration for clinical translation is the question of how to deliver hCSSCs to human cornea. Here, cells were applied topically in a fibrin gel after epithelial debridement, enabling cell contact with stromal ECM. This method aligns with existing ophthalmic techniques using controlled epithelial removal. Fibrin-based biomaterials, already common as biodegradable sealants and scaffolds in eye surgery, offer rapid biodegradation and good ocular compatibility, making them suitable for temporary cell delivery. Clinically, hCSSCs in a Good Manufacturing Practices (GMP)-grade fibrin droplet, such as TISSEEL, could be placed on the de-epithelialized corneal surface to promote local cell–matrix interaction during early remodeling.

Immune compatibility is also an important issue. hCSSCs, like mesenchymal stromal cells, have immunomodulatory features such as the low expression of major histocompatibility complex (MHC) class II molecules and the secretion of anti-inflammatory mediators (e.g., TGFβ3). Combined with the cornea’s immune privilege, these features suggest allogeneic hCSSC therapy may work without systemic immunosuppression. However, immune reactions in inflamed or vascularized corneas could affect cell survival and treatment success. Studies in larger animals are needed to assess immune responses and how long cells must persist for effective remodeling.

Recent advances in cell therapy for corneal opacity includes the use of corneal stromal keratocytes (CSKs). In a rat model of irregular phototherapeutic keratectomy, the intrastromal injection of hCSKs demonstrated a modest reduction of chronic haze [50]. CSKs are featured for native ECM production and stromal reconstruction, expressing high levels of stromal collagens and keratocan [13]. In contrast, hCSSCs exhibit stronger immunomodulatory and anti-fibrotic properties [9,17,18]. Our data thus indicate that hCSSCs are particularly effective at destabilizing the fibrotic niche, whereas CSKs are better suited for structural matrix rebuilding. These complementary properties support a staged therapeutic paradigm: initial hCSSC-mediated fibrosis resolution and microenvironmental normalization, followed by hCSK-driven architectural reconstruction. Such combinatorial strategies may ultimately achieve more complete restoration of native-like stromal architecture, hence benefiting transparency and light passage. This innovative concept has been proven through a combined treatment involving topical CSSC treatment followed by intrastromal CSK injection at one week after acute injury to achieve a greater efficacy in scar inhibition and maintaining corneal clarity [13].

A major barrier in evaluating chronic scar treatment is the inter-corneal variability. Even standardized injury produces heterogeneous opacity severity due to inherent biological differences in wound response across corneas, hence complicating end-point comparisons. We addressed this challenge by developing a reproducible double injury model that generates moderate, persistent scarring with reduced variability. This model more closely approximates clinical scenarios involving recurrent stromal injury [51]. In our optimized protocol, one-week post-double injury created moderate scarring consistently with narrow coefficient of variation (~36%) and expression of typical fibrosis-associated genes. We further selected corneas with a detectable opacity of 5% or more for evaluating CSSC treatment efficacy, to ensure a consistent sampling.

Equally important, we implemented longitudinal scar volumetric quantification using OCT coupled with dual-threshold computational segmentation. The inter-corneal variability of scar severity makes it difficult to perform reliable analyses based on grouping or comparing different corneas, as baseline differences may confound the interpretation of treatment outcomes. Consequently, a more robust approach is to evaluate changes within the same cornea over time, allowing each cornea to serve as its own control. This longitudinal assessment necessitates non-invasive examinations, which enable repeated evaluation of scar changes at multiple time points, rather than end-point assessment. OCT offers the ability to conduct longitudinal assessment of the same region. Development of our processing was influenced by several factors as corneal volumes can be large and require more time for processing than single axial images. Nevertheless, volumetric imaging is the only way to accurately measure scar volume in the cornea. To manage time and computing resources, we chose to restrict our field used in the analysis to the anterior 2/3 of the cornea. This field was large enough to encompass the wound site, and it provided a more consistent signal within the area being analyzed by avoiding the characteristic falloff found in OCT images as the tissue presents further away from the scan head. This stability of signal also allows us to determine set threshold levels which were applied consistently in the segmentation. We employed a dual threshold strategy to distinguish between the total corneal and fibrotic volumes. We set the first fixed threshold to reveal the corneal button and total corneal volume. Scar volumes were then determined by examining the normal intensity levels in over 50 naïve corneas. Signals in regions higher than those found in naïve corneas define the threshold boundary and this setting identifies regions of increased optical density, which represents the hyperreflective scar volume. Generally speaking, this approach enables within-eye comparison over time and minimizes confounding factors from baseline heterogeneity and increases sensitivity to dynamic remodeling. This also allows for the use of fewer animals to provide a sufficient sample size to achieve reliable results. Hence, quantitative opacity volume provides a robust surrogate marker for fibrotic scarring and establishes a framework for future mechanistic and therapeutic studies. Integration of automated or machine learning-based segmentation may further enhance analytical precision.

Several limitations warrant consideration. The corneal injury involved anterior stromal ablation of limited depth and size, which does not fully replicate deeper, infectious, or chemical injuries encountered clinically. Chronic scars likely differ from acute haze in ECM cross-linking density, cellular senescence, immune composition, and biomechanical stiffness. These factors may influence the responsiveness to therapy. Multiple dosing may enhance the therapeutic efficacy; however, epithelial debridement is required to open the stroma for cell application and repeated debridement could compromise the regeneration and integrity of epithelial basement membrane, potentially leading to unforeseen adverse effects. Alternative delivery strategies, including minimally invasive stromal injection or cell-free approaches using MSC- or hCSSC-derived exosomes, warrant investigation. Given that hCSSC exosomes deliver miR-29a [18], which modulates MMP-related pathways [52,53], cell-free platforms may preserve anti-fibrotic efficacy while simplifying the regulatory hurdles in clinical translation.

## 5. Conclusions

This study has established that hCSSC therapy actively reduces pre-existing corneal fibrosis, associated with a shift in proteolytic balance toward increased MMP2/TIMP2 ratio and enhanced matrix remodeling. By demonstrating that chronic stromal scar tissue is biologically modifiable, we redefine the therapeutic potential of corneal regenerative cell therapy. Coupled with a reproducible chronic scarring model and quantitative longitudinal imaging platform, these findings provide both mechanistic insight and translational momentum toward the non-surgical treatment of chronic corneal opacities.

## Figures and Tables

**Table 1:** **Intra-class correlation analysis of scar volume measurement**. Percentages of scar volume over the entire corneal volume were obtained for 12 mouse corneas with varied levels of opacities. Image processing and scar volume measurements were performed by two masked raters (I and II), and the entire procedure was repeated after 24 h. Two-way random effects model, where both people effects and measures effects are random, was used. Both (**a**) inter- and (**b**,**c**) intra-rater reliabilities were >0.9 (excellent), with the confidence interval close to 1.00. ^a^ Type A intraclass correlation coefficients using an absolute agreement definition. df—degree of freedom.

(a) Inter-Rater Variability							
	Intraclass Correlation	95% Confidence Interval		F Test with True Value 0			
		Lower Bound	Upper Bound	Value	df1	df2	Sig
Single Measures	0.998 ^a^	0.995	1.000	1236.7	11	11	0.000
Average Measures	0.999	0.997	1.000	1236.7	11	11	0.000
(b) Intra-Rater I Variability							
	Intraclass Correlation	95% Confidence Interval		F Test with True Value 0			
		Lower Bound	Upper Bound	Value	df1	df2	Sig
Single Measures	0.994 ^a^	0.978	0.998	358.25	11	11	0.000
Average Measures	0.997	0.989	0.999	358.25	11	11	0.000
(c) Intra-Rater II Variability							
	Intraclass Correlation	95% Confidence Interval		F Test with True Value 0			
		Lower Bound	Upper Bound	Value	df1	df2	Sig
Single Measures	1.000 ^a^	0.999	1.000	7342.34	11	11	0.00
Average Measures	1.000	1.000	1.000	7342.34	11	11	0.00

## Data Availability

The original contributions presented in this study are included in the article/Supplementary Materials. Further inquiries can be directed to the corresponding author.

## Supplementary Materials

The following supporting information can be downloaded at https://www.mdpi.com/article/10.3390/cells15070615/s1, Figure S1: A mouse model of corneal anterior stromal injury and CSSC treatment; Figure S2: Experimental setup and timeline of the chronic corneal scarring model; Figure S3: Representative corneal images showing development of chronic corneal scarring model; Figure S4: Human corneal stromal stem cell (CSSC) characterization; Figure S5: The anti-scarring outcome of CSSC treatment in acute corneal wounds; Figure S6: HC436 treatment on chronic corneal scarring; Figure S7: HC439 treatment on chronic corneal scarring; Figure S8: HC728 treatment on chronic corneal scarring; Figure S9: Representative images of untreated scarred corneas; Figure S10: Representative images of scarred corneas treated with fibrin gel only; Figure S11: Whole-mount immunofluorescence of fibrosis marker expression in mouse corneas at PTW3 of CSSC treatments and controls; Figure S12: Validation of in vivo OCT morphometry with immunohistochemistry; Table S1: Donor cornea information; Table S2: Expression primers used in this study; Table S3: Antibodies used in this study; Table S4: CSSC viability under fibrin gel encapsulation at time intervals; Table S5: Total MMP activity in intracellular (cell lysates) and extracellular (culture supernatants) fractions of human CSSC treated with M1 pro-inflammatory RAW conditioned media.

## Abbreviations

The following abbreviations are used in this manuscript:

ALDH	Aldehyde dehydrogenase
αSMA	α-smooth muscle actin
CCT	Central corneal thickness
Col	Collagen
CoV	Coefficient of variation
CSK	Corneal stromal keratocytes
CSSC	Corneal stromal stem cell
EC	Extracellular
ECM	Extracellular matrix
Fn	Fibronectin
IC	Intracellular
MMP	Matrix metalloproteinase
MyoF	Myofibroblast
OCT	Optical coherence tomography
OVM	Opacity volume measurement
PTW	Post-treatment week
SF	Stromal fibroblast
TGFβ	Transforming growth factor β
TIMP	Tissue inhibitor of matrix metalloproteinase
