# Supplementary material for: Human Corneal Stromal Stem Cell Treatment Reduces Established Opacities in Chronic Corneal Scarring
Source: Cells. 2026 Mar 30;15(7):615. doi: 10.3390/cells15070615 (PMC13072307; doi:10.3390/cells15070615)
Supplement: Supplementary file 1 [file cells-15-00615-s001.zip › cells-4207647-supplementary.pdf]

Human corneal stromal stem cell treatment reduced chronic corneal opacities

Supplementary Information

Supplementary Methods

Clonal expansion and colony-forming efficiency

Cells were seeded at 5 cells/cm^2 in a tissue culture plate (100-mm diameter), i.e., 400 cells per dish. Fresh medium was replenished every 3 to 4 days. At day 10, cells were fixed with ice-cold methanol for 10 min. After PBS washes, the cells were stained with 0.1% crystal violet in 20% methanol for 5 min. After rinses with distilled water and brief drying, the entire plate was photographed and clones with >50 cells were quantified. The colony-forming efficiency was calculated by the number of colonies over the number of cells seeded.

Cell growth assay by xCelligence

The cell growth efficiency was evaluated using xCelligence real-time cell analyzer RTCA SP (Agilent/ACEA, Santa Clara, CA). Cells were seeded at a density of 4x103 cells in each well of an E-plate 96, following the instructions of manufacturer. The impedance value of each well was automatically recorded every 15 min for a period of 150 hours and expressed as a Cell Index (CI) value. The experiment was conducted in triplicate. The cell doubling time was calculated at the log phase using the RTCA software (Agilent).

Flow cytometry

Cells were collected after trypsinization and passed through a 70-μm cell strainer and a 40-μm cell strainer sequentially to obtain single cell suspension. After blocking with Fc-Block (BioLegend), the cells were incubated with primary antibody (Supplementary Table S2) for 30 min on ice in dark. After washes, the cells were fixed with 1% paraformaldehyde (Sigma-Aldrich) before analysis using Cytoflex LX (Beckman). The percentage of positively labeled cells was calculated following the manufacturer's instructions.

Trypan blue dye exclusion assay

Single cell suspension (10 μl) was mixed with the same volume of trypan blue (0.4%, Sigma-Aldrich) and allow to incubate for 1 min. The mixture (10 μl volume) was applied to the

Countess chamber slide. Viable cell quantity and percentage of viability were obtained using the Countess automated cell counter (Invitrogen).

Supplementary Table S1. Donor cornea information

Lab no.	Age	Gender	Cause of Death	Days in Optisol preservation
**436**	33	M	Multiple cerebral infarcts	7
**439**	57	F	Anoxia brain injury	9
**728**	55	M	Gun shot	8

Supplementary Table S2. Expression primers used in this study

Gene Name	Primer Sequences	GenBank Accession No.	Amplicon size
**Mouse genes**			
**COL3A1**	Forward: CGTAAGCACTGGTGGACAGA<br>Reverse: CGGCTGGAAAGAAGTCTGAG	NM_009930.2	155 bp
**αSMA**	Forward: CTCTGCCTCTAGCACACAACT<br>Reverse: ACGCTCTCAAATACCCCGTTT	NM_007392.3	332 bp
**18s**	Forward: GTGCATGGCCGTTCTTAGTT<br>Reverse: ATGCCAGAGTCTCGTTCGT	NR_003278.3	69 bp
**Human genes**			
**MMP2**	Forward: GGCCCTGTCACTCCTGAGAT<br>Reverse: GGCATCCAGGTTATCGGGGA	NM_004530.5	473 bp
**MMP9**	Forward: TGCCAGTTTCCATTCATCTTCC<br>Reverse: CTGCGGTGTGGTGGTGGTT	NM_004994.3	518 bp
**TIMP1**	Forward: TTTGAGTCCGGTGGACGATG<br>Reverse: TTGTCGGCGATAAGGAAGGG	NM_003254.2	152 bp
**TIMP2**	Forward: AAGCGGTCAGTGAGAAGGAAG<br>Reverse: GGGGCCGTGTAGATAAACTCT	NM_003255.3	198 bp
**18s**	Forward: CCCTGTAATTGGAATGAGTCC<br>Reverse: GCTGGAATTACCGAGGCT	NR_145820.1	69 bp

Supplementary Table S3: Antibodies used in this study

Antibody (clone)	Sources	Working concentrations
Anti-αSMA-AlexaFluor488 (1A4)	Invitrogen 53-9760-82	0.5 μg/ml
Rabbit anti-mouse fibronectin FN	Abcam AB2413	0.2 μg/ml
PE anti-human CD31 conjugate	BioLegend 303105	5 μl per 10^6 cells
FITC anti-human CD45 conjugate	BioLegend 304005	5 μl per 10^6 cells
APC anti-human CD73 conjugate	BioLegend 344006	5 μl per 10^6 cells
Anti-human ALDH3A1 (Aldehyde dehydrogenase 3A1)	Proteintech 15578-1-AP	3-5 μg/ml
AlexaFluor488 AffiniPure Donkey anti-mouse IgG (H+L)	Jackson ImmuoRes Lab 715-545-150	0.5 μg/ml
AlexaFluor594 AffiniPure Donkey anti-rabbit IgG (H+L)	Jackson ImmuoRes Lab 711-585-152	0.5 μg/ml
APC mouse IgG1, κ isotype antibody	BioLegend 400120	5 μl per 10^6 cells
FITC mouse IgG1, κ isotype antibody	BioLegend 400108	5 μl per 10^6 cells
PE rat IgG2a, κ isotype antibody	BioLegend 400521	5 μl per 10^6 cells
DAPI (4',6-Diamidine-2'-phenylinodole dihydrochloride)	Roche 10236276001	1-2 ng/ml

Supplementary Table S4. CSSC viability under fibrin gel encapsulation at time intervals

**Time**	**1h**	**2h**	**4h**	**8h**	**12h**	**24h**	**36h**	**48h**
**Viability (%)**	97.3±2.4	97.1±1.9	97±2.5	96.8±2.4	95.3±3.4	93.6±4.3	90.3±6.3	86.2±5.3

Supplementary Table S5. Total MMP activity in intracellular (cell lysates) and extracellular (culture supernatants) fractions of human CSSC treated with M1 pro-inflammatory RAW conditioned media. Controls were M0 RAW conditioned media and sham media. Data are presented in mean ± SD. # MMP activity in U/mg total protein. * $P<0.05$ comparing between M0 and M1 treatments; non-parametric one-way ANOVA Mann-Whitney U test.

Cell and treatments	Intracellular MMP activity #	Extracellular MMP activity #	Total MMP activity #	% Extracellular/ Total activity
**HC436**				
**Sham medium**	93.3±4.7	12±0.6	105.3±5.3	11.4±0.6
**M0**	110.5±5.5	20.8±1 *	131.3±6.6	15.8±0.8 *
**M1**	128.6±6.4	30.1±1.5 *	158.8±7.9	19±1 *
**HC439**				
**Sham medium**	25.2±1.3	11.2±0.6	36.4±1.8	30.6±1.5
**M0**	28±1.4	13.5±0.7	41.5±2.1	32.5±1.7
**M1**	28.2±1.4	14±0.7	42.2±2.1	33.1±1.6
**HC728**				
**Sham medium**	15.3±0.7	3.72±0.2	19±0.9	19.6±1
**M0**	33±1.7	14.1±0.7 *	47.1±5.4	30±1.7 *
**M1**	30.2±1.5	25.8±3.5 *	56±5.1	46.1±3.4 *

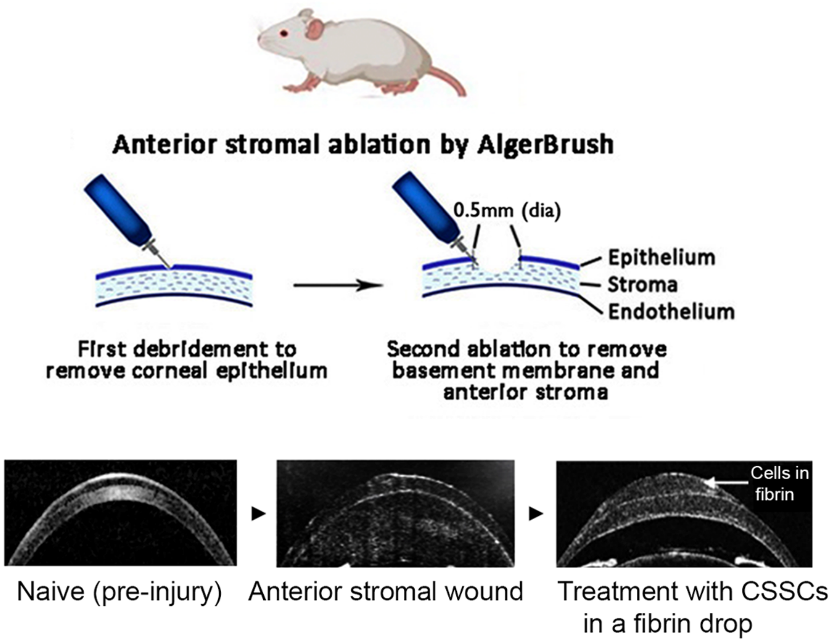

Supplementary Figure S1. A mouse model of corneal anterior stromal injury and CSSC treatment. Using an Algerbrush mechanical burring to create an epithelial wound (2 mm in diameter), a second ablation injury disrupted the Bowman's membrane and produced anterior stromal wound of 0.5 mm diameter and 20-30 μm depth. After saline washes, the corneas received topical Tobramycin thrice daily for 3 days. Multiple injuries were similarly performed at consecutive weeks.

On the day of treatment, the corneal epithelium was removed. After washes, human CSSCs were applied in a fibrin gel (a mix of fibrinogen and thrombin). The cell-treated corneas received TobraDex thrice daily in the first week, then twice daily in the second week, and once daily in the third week.

OCT images at the bottom panel show the cross-sectional cornea before and after injury as well as post-treatment with cells/fibrin drop covering the wound region.

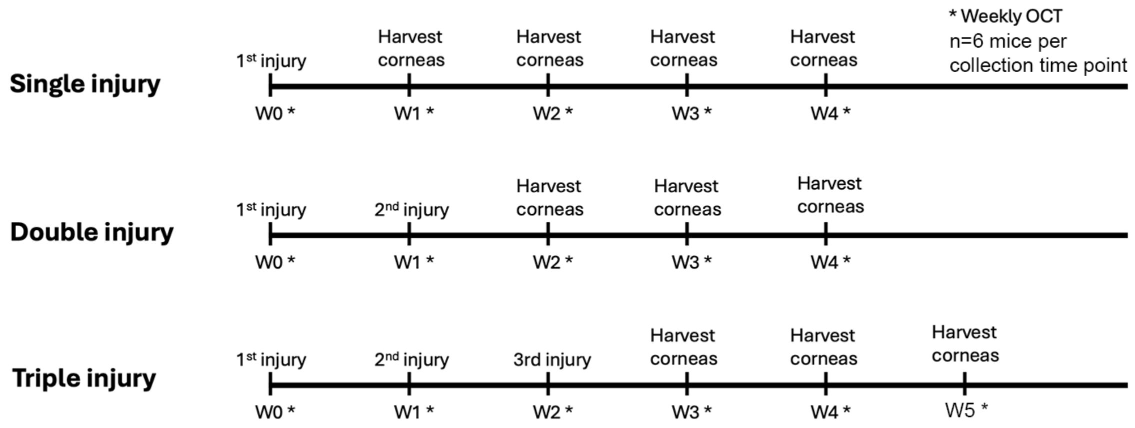

Supplementary Figure S2. Experimental setup and timeline of the chronic corneal scarring model. Mouse corneas received single, double, or triple injury at consecutive weeks. Corneas were harvested weekly at 1 to 4 weeks to reveal the scarring outcomes.

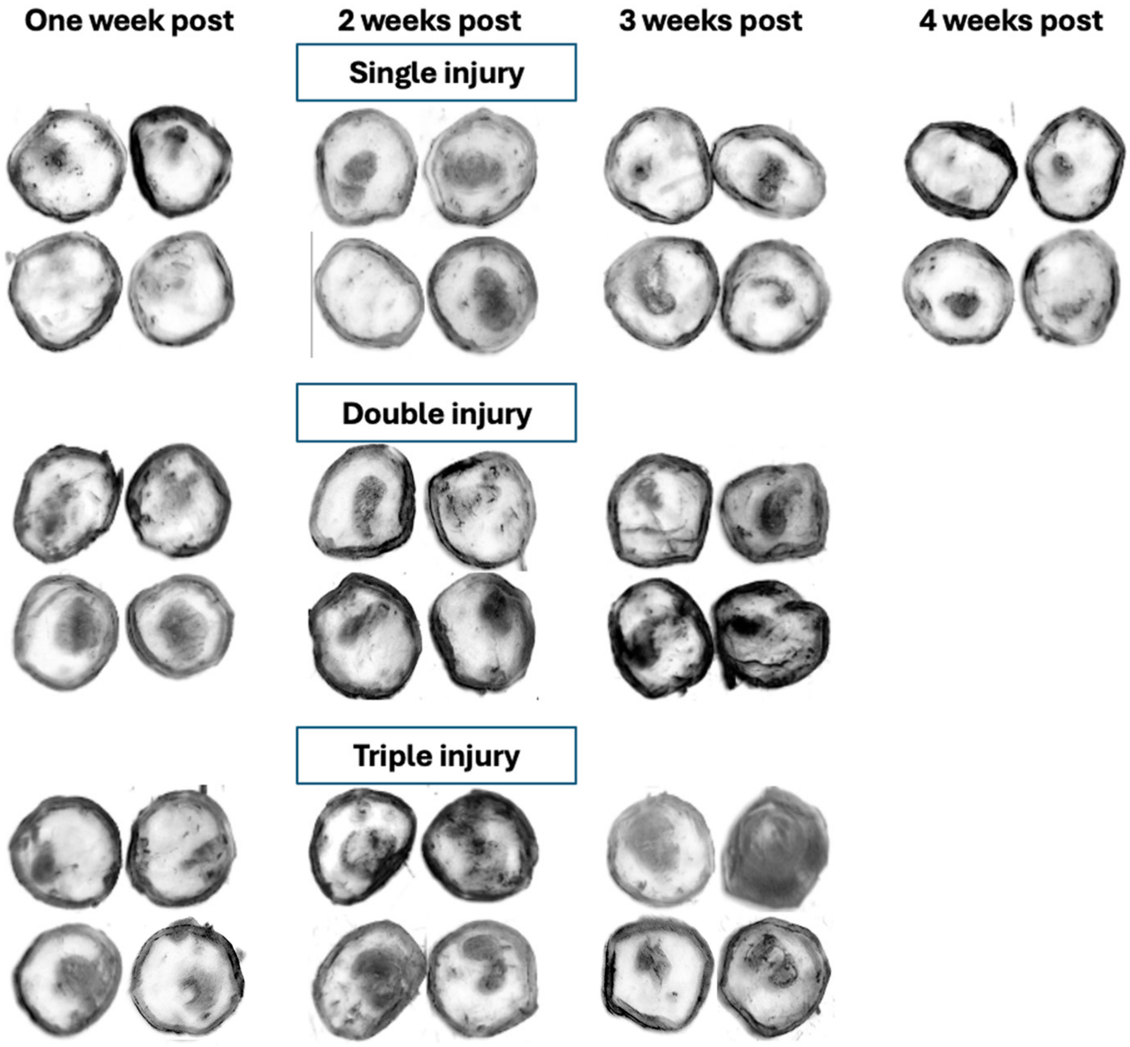

Supplementary Fig. S3. Representative corneal images showing development of chronic corneal scarring model. Mouse corneas received anterior stromal injury caused by single mechanical ablation at time 0 or multiple ablations at consecutive weeks. The corneas were collected at different weeks post last injury to examine the efficiency of scar formation (the percentage of scarring area and expression of fibrosis-related genes).

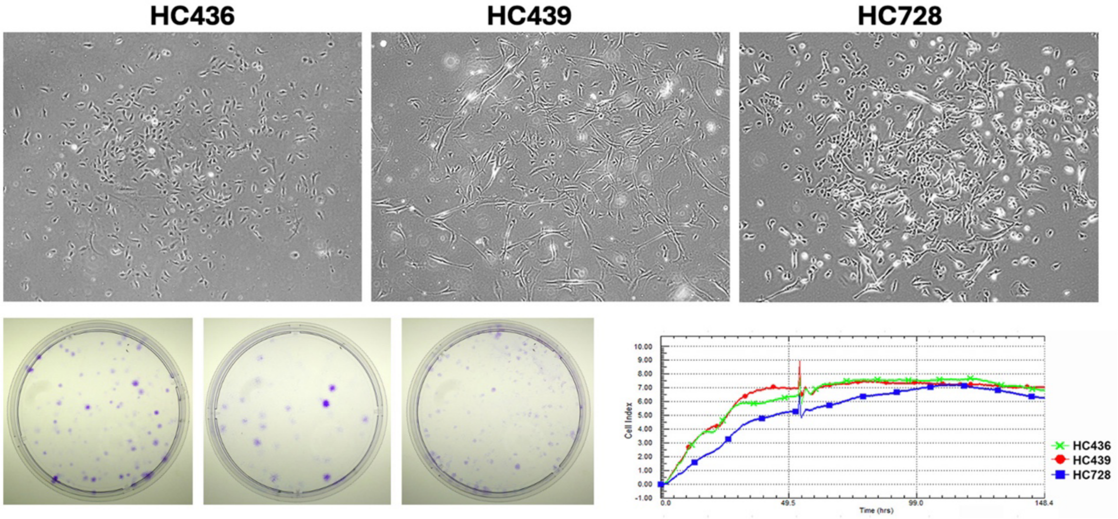

	CD73	ALDH3A1	CD31	CD45
**HC436**	95.5%	35%	0%	0%
**HC439**	94.8%	47%	0.12%	0.13%
**HC728**	96.3%	22.7%	0%	0.7%

Supplementary Fig. S4. Human corneal stromal stem cell (CSSC) characterization. (**A**) Phase contrast micrographs of HC436, HC439, and HC728 cells at passage 2 (P2). (**B**) Clonal proliferation of CSSCs at 10 days after seeding at 600 cells per 100-mm tissue culture dish. Cell colonies were stained with crystal violet and colonies with >50 cells were quantified to calculate the colony-forming efficiency. (**C**) Cell growth examination by xCelligence. (**D**) By flow cytometry analysis, CSSCs exhibited $CD73^{high}$ $ALDH3A1^{+}$ $CD31^{neg}$ $CD45^{neg}$ phenotypically.

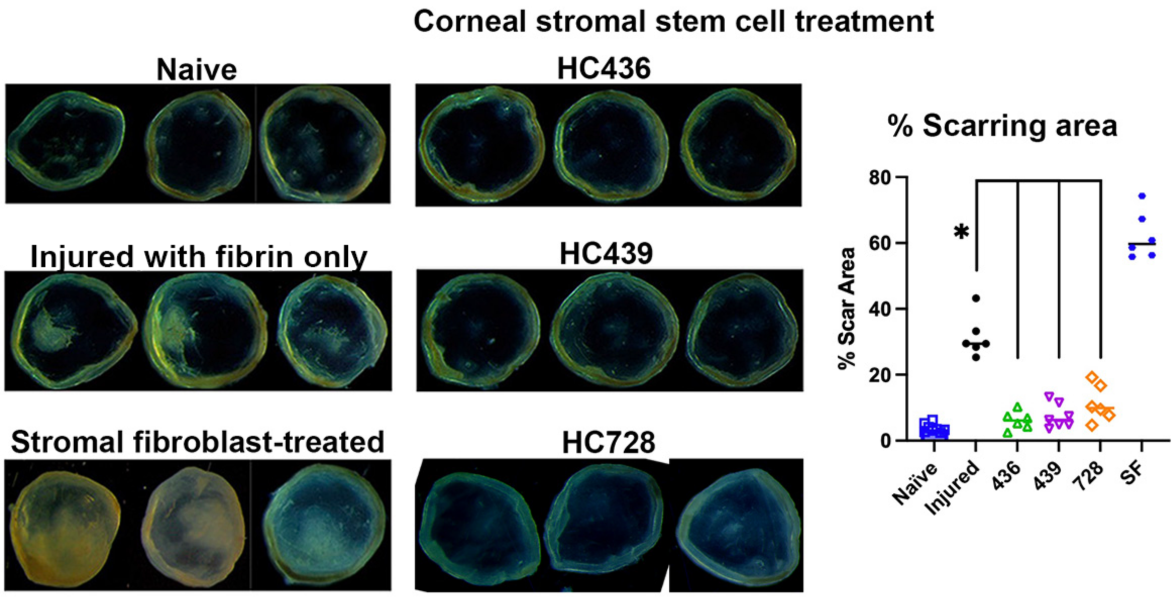

Supplementary Fig. S5. The anti-scarring outcome of CSSC treatment in acute corneal wounds. Compared to naïve corneas, the corneas with anterior stromal wound developed intense opacity at 2 weeks post-injury. Topical treatment with CSSC (HC436, HC439, and HC728) at 5×10^4 cells in a drop of fibrin gel per application inhibited more than 50% scarring. In contrast, wounds treated with stromal fibroblasts elevated the scarring outcome. The scarring area and the entire corneal area were obtained using ImageJ (FIJI) measurement for the calculation of scarring percentages. Significant scar inhibition was noted after treatments with these CSSC batches. * $P<0.05$ compared to injured corneas, non-parametric one-way ANOVA. Detailed results were reported in Santra et al. 2024.

Santra, M. *et al.* Good manufacturing practice production of human corneal limbus-derived stromal stem cells and in vitro quality screening for therapeutic inhibition of corneal scarring. *Stem Cell Res Ther* **15**, 11 (2024).

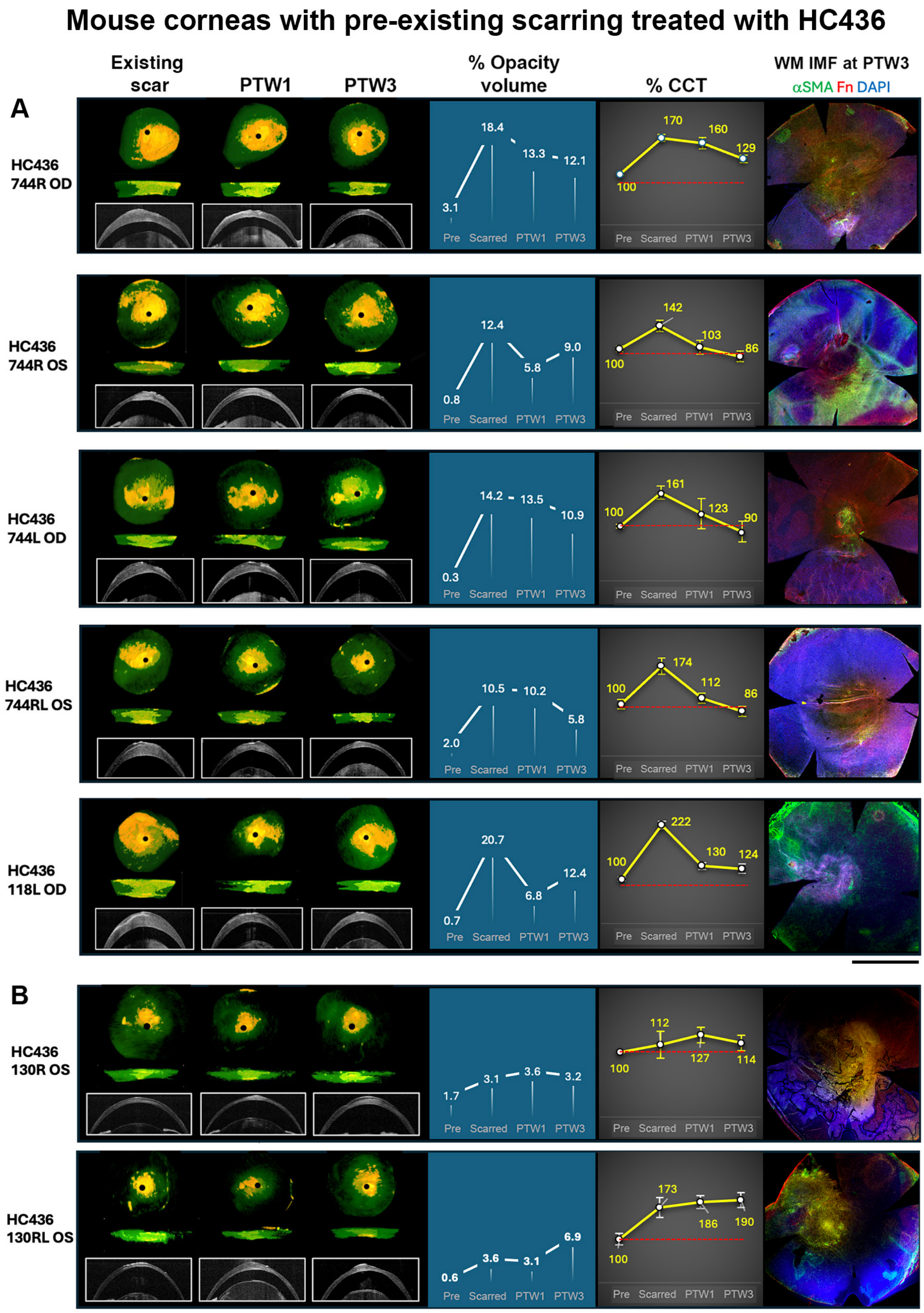

Supplementary Fig. S6. HC436 treatment on chronic corneal scarring. (**A**) Five of 7 corneas with pre-existing scarring exhibited a progressive scar volume reduction. Central corneal thickness (CCT) returned to pre-injury (naïve) levels (red dotted lines). End point analysis with whole-mount immunofluorescence (WM IMF) showed a moderate expression of fibrosis markers (α-smooth muscle actin αSMA and fibronectin Fn). (**B**) Two remaining corneas did not reveal any opacity reduction after HC436 treatments. Scale bar: 1 mm.

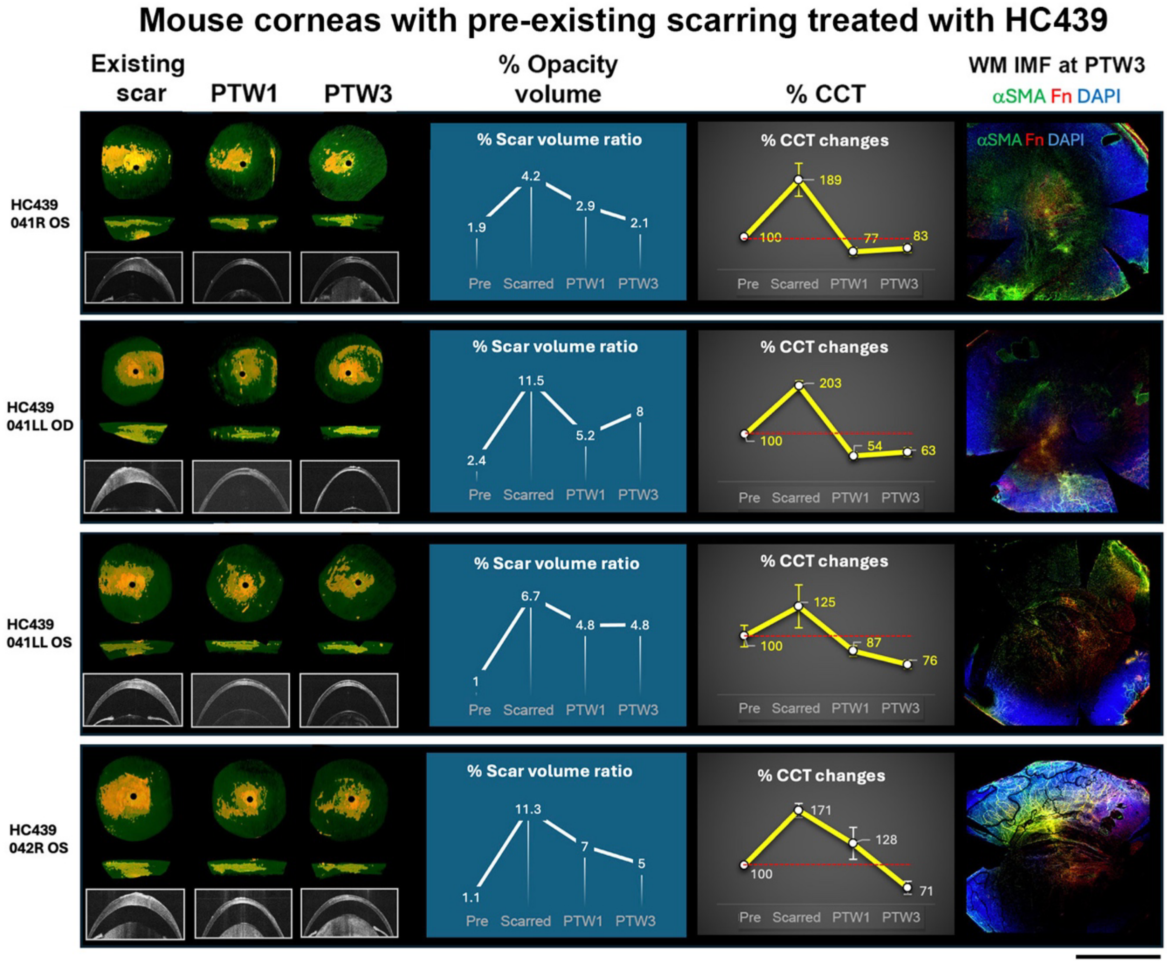

Supplementary Fig. S7. HC439 treatment reduced chronic corneal scarring. Mouse corneas with pre-existing scarring exhibited a progressive scar volume reduction after cell therapy. Central corneal thickness (CCT) returned to pre-injury (naïve) levels (red dotted lines). End point analysis with whole-mount immunofluorescence (WM IMF) showed a moderate expression of fibrosis markers (α-smooth muscle actin αSMA and fibronectin Fn). Scale bar: 1 mm.

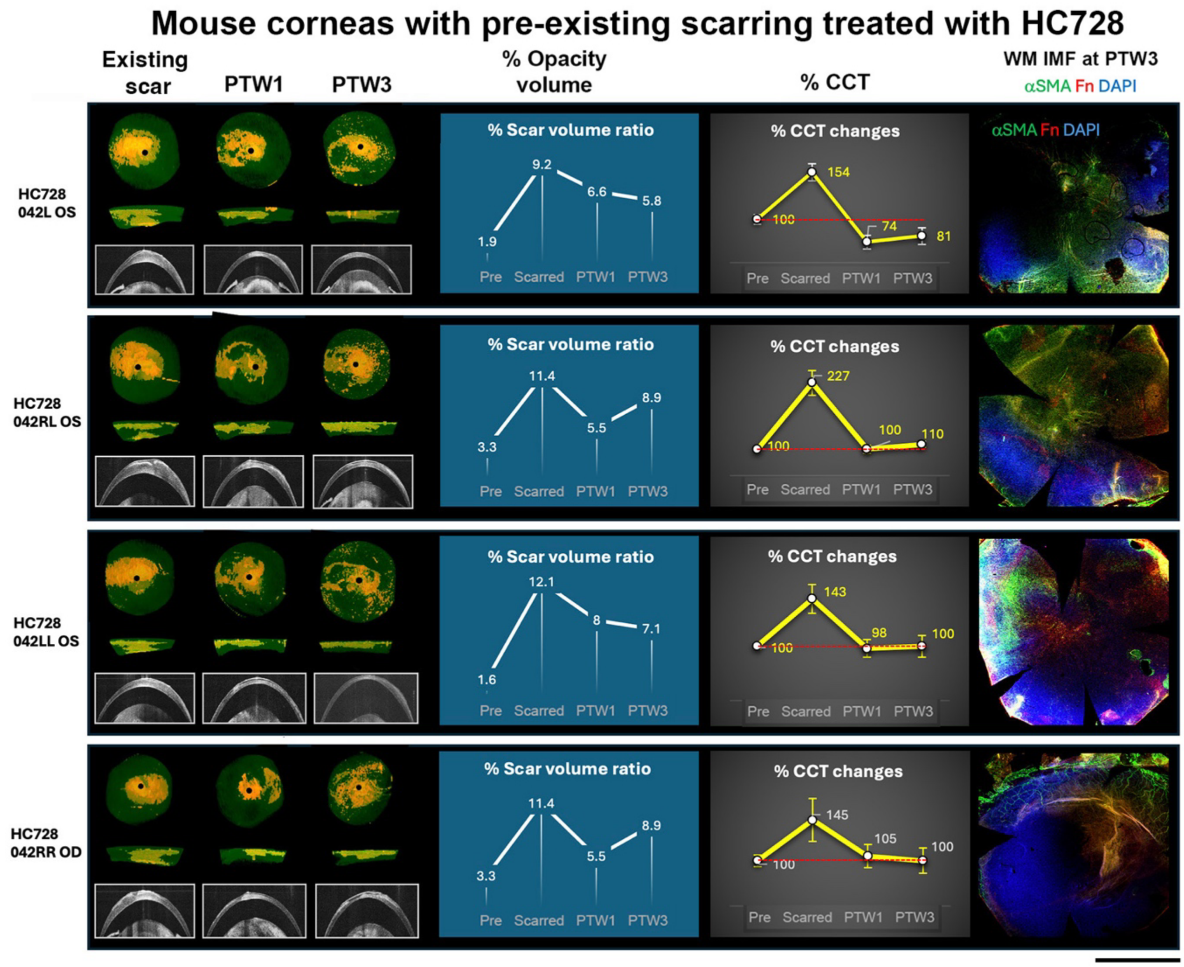

Supplementary Fig. S8. HC728 treatment reduced chronic corneal scarring. Mouse corneas with pre-existing scarring exhibited progressive scar volume reduction after cell therapy. Central corneal thickness (CCT) returned to pre-injury (naïve) levels (red dotted lines). End point analysis with whole-mount immunofluorescence (WM IMF) showed a moderate expression of fibrosis markers (α-smooth muscle actin αSMA and fibronectin Fn). Scale bar: 1 mm.

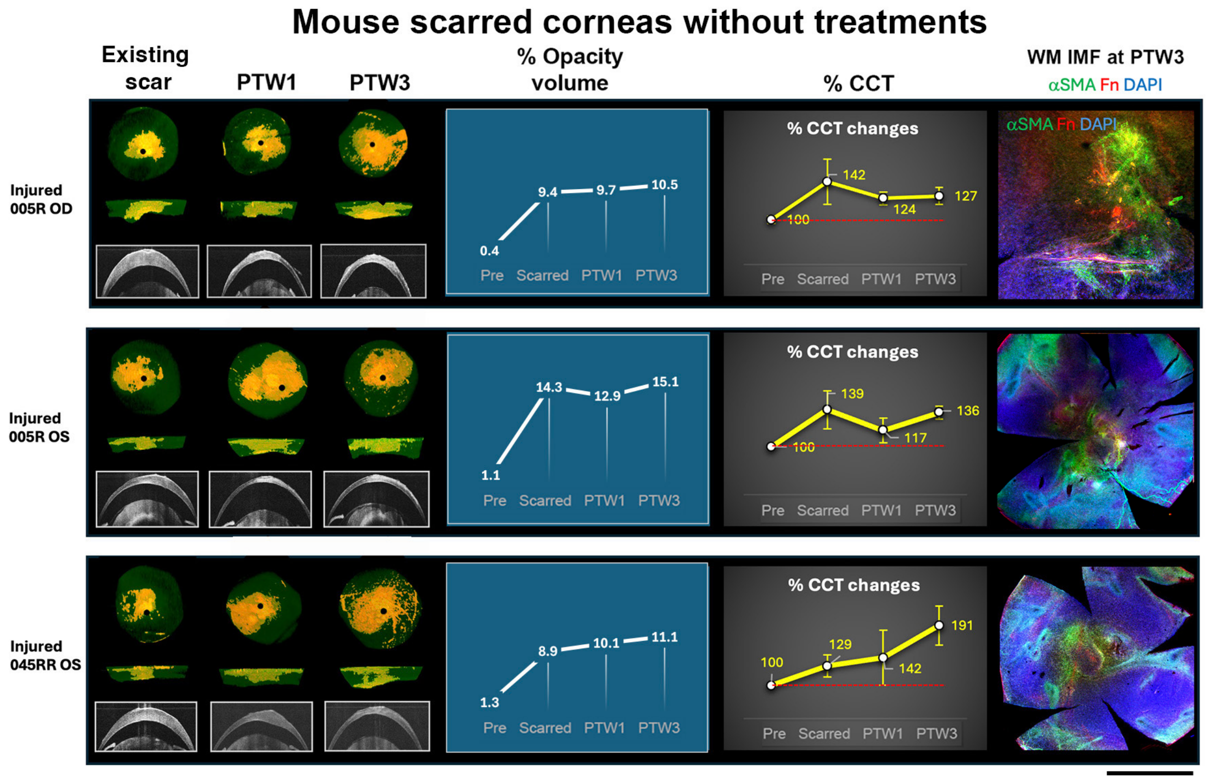

Supplementary Fig. S9. Representative images of untreated scarred corneas. Both opacity volume and central corneal thickness (CCT) were consistent at higher levels, indicating of stable scarring. End point analysis with whole-mount immunofluorescence (WM IMF) showed extensive expression of fibrosis markers (α-smooth muscle actin αSMA and fibronectin Fn). Scale bar: 1 mm.

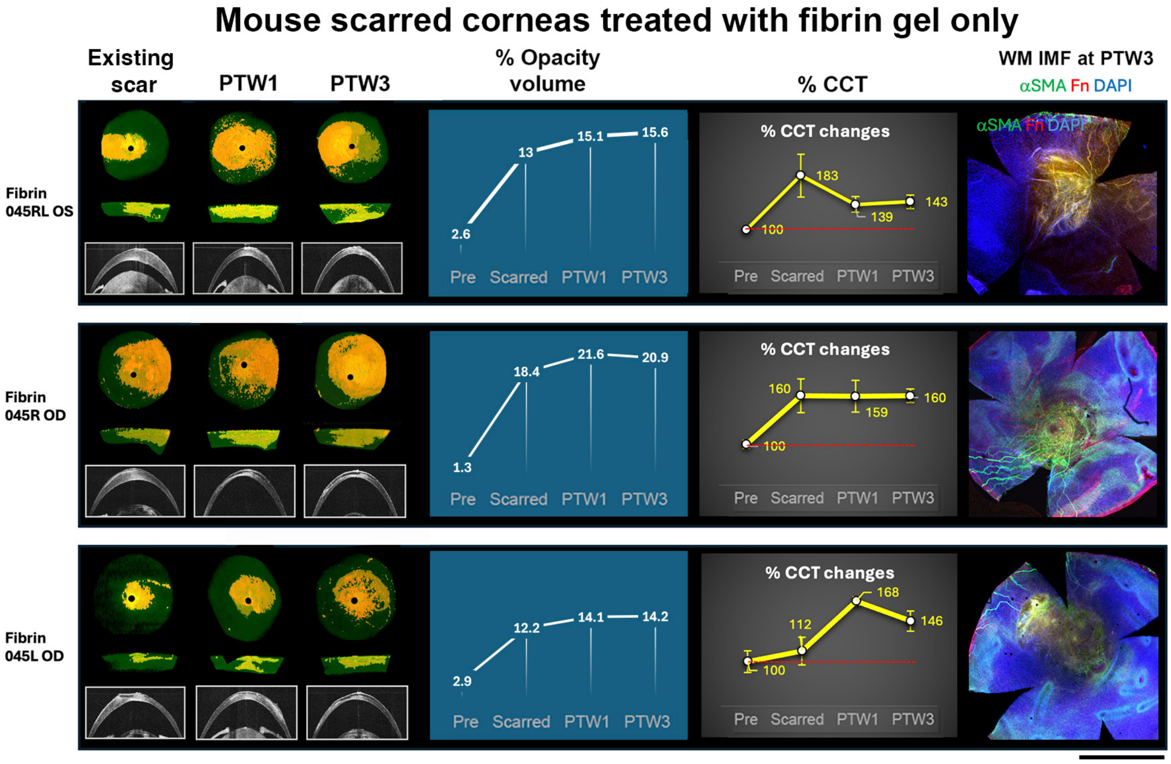

Supplementary Fig. S10. Representative images of scarred corneas treated with fibrin gel only. Both opacity volume and central corneal thickness (CCT) remained at higher levels. End point analysis with whole-mount immunofluorescence (WM IMF) showed extensive expression of fibrosis markers (α-smooth muscle actin αSMA and fibronectin Fn). Scale bar: 1 mm.

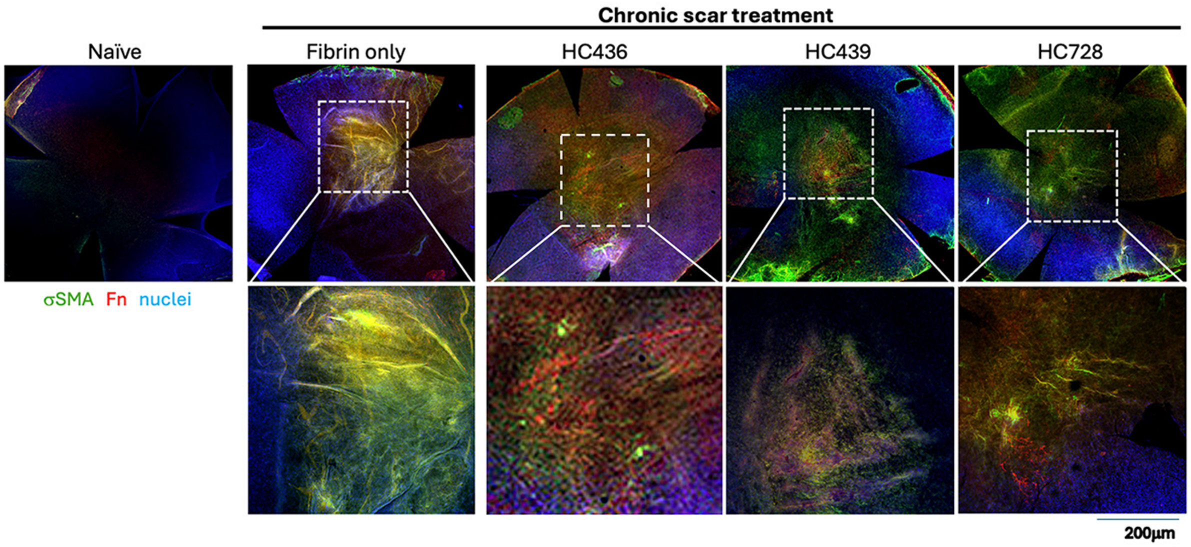

Supplementary Fig S11. Whole-mount immunofluorescence of fibrosis marker expression in mouse corneas at PTW3 of CSSC treatments and controls. The confocal images showed strong expression of fibrosis markers, αSMA and Fn, in the injured corneas with fibrin gel only, in contrast to the naïve cornea. Corneas treated with CSSC (HC436, 439, and 728) exhibited a consistently lower expression of both markers.

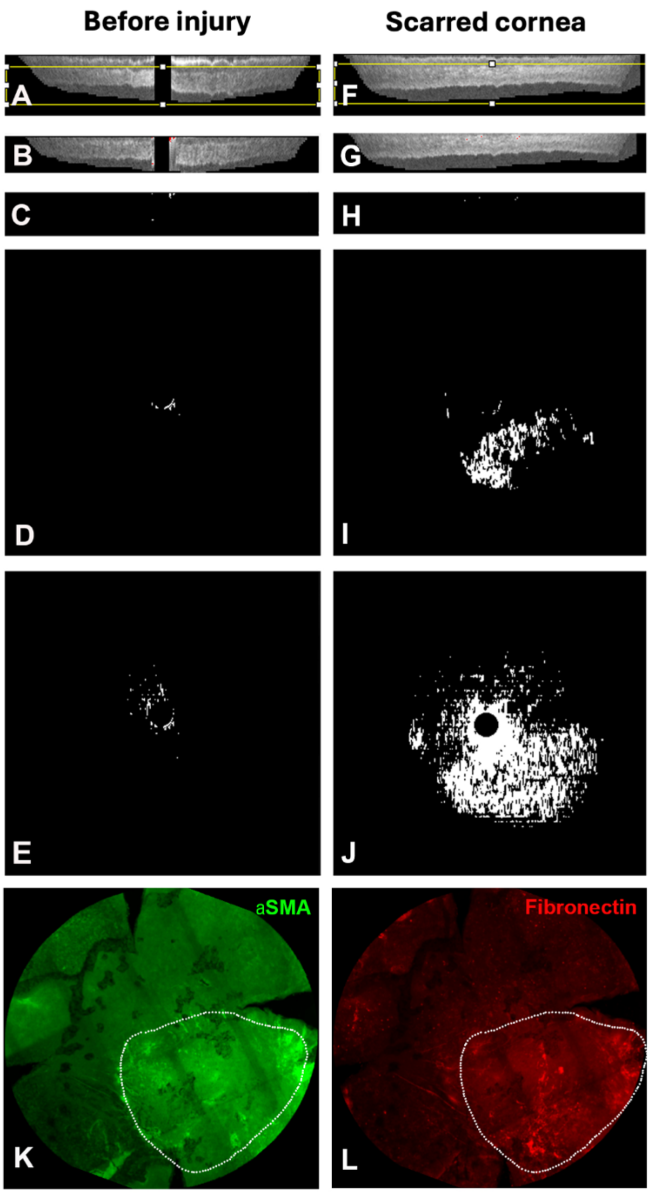

Supplementary Fig S12. Validation of *in vivo* OCT morphometry with immunohistochemistry. The threshold cutoff for detecting fibrotic signal in a scarred cornea is determined using OCT volumes segmented from the same cornea in its pre-wound (naïve) state (**left column**). The flattened OCT volume is opened, and stromal volume is selected (**A-B**). This stromal volume is thresholded to identify the baseline signal level in the naive stroma, with threshold values adjusted to retain only a minimal number of voxels. The thresholded volume is converted into a binary mask (**C**), and resliced to obtain coronal view (**D**). A Z-projection of this volume reveals the overall distribution of signal throughout the stroma (**E**). The same processing is applied to the OCT volume acquired from same cornea with scarring caused by injury (**right column**). Importantly, the same threshold cutoff determined from the naïve state is reused without modification (**F** to **I**). Because OCT detects structures based on changes in refractive index, dense scar tissue produces stronger (brighter) signals (**I**). Thus,

any signal remaining after applying the naïve-derived threshold in the scarred cornea is interpreted as fibrotic (**J**).

To validate the presence and spatial distribution of fibrosis, the same cornea was harvested, fixed, and subjected to immunohistochemistry (IHC). The sample was labeled for αSMA (a marker for myofibroblasts, **K**) and fibronectin (a fibrosis-associated ECM protein, **L**), Both are absent in the naïve cornea. Whole cornea was imaged using an Olympus FV4000 confocal microscope with a 10x apochromatic objective (NA 0.40), and stitched image volume was Z-projected to visualize overall signal patterns. Images were adjusted for display.

The OCT-detected fibrosis signal is highly corresponding to the IHC signals on the same corneal tissue (**J**-**L**). It is important to note that OCT imaging was performed *in vivo,* whereas IHC was conducted on *ex vivo* wholemount tissue with flattening of cornea prior to confocal imaging; therefore, exact spatial correspondence is not expected. In addition, chronic scar primarily composed of accumulated and disfigured ECM and collagen with limited cellular components, including fibroblasts and myofibroblasts. Hence, we anticipate a general spatial agreement rather than exact overlap between OCT-derived fibrotic regions and IHC signals.
